# A chitinase with two catalytic domains is required for organization of the cuticular extracellular matrix of a beetle

**DOI:** 10.1371/journal.pgen.1007307

**Published:** 2018-03-28

**Authors:** Mi Young Noh, Subbaratnam Muthukrishnan, Karl J. Kramer, Yasuyuki Arakane

**Affiliations:** 1 Department of Applied Biology, Chonnam National University, Gwangju, South Korea; 2 Department of Biochemistry and Molecular Biophysics, Kansas State University, Manhattan, Kansas, United States of America; The University of North Carolina at Chapel Hill, UNITED STATES

## Abstract

Insect cuticle or exoskeleton is an extracellular matrix formed primarily from two different structural biopolymers, chitin and protein. During each molt cycle, a new cuticle is deposited simultaneously with degradation of the inner part of the chitinous procuticle of the overlying old exoskeleton by molting fluid enzymes including epidermal chitinases. In this study we report a novel role for an epidermal endochitinase containing two catalytic domains, TcCHT7, from the red flour beetle, *Tribolium castaneum*, in organizing chitin in the newly forming cuticle rather than in degrading chitin present in the prior one. Recombinant TcCHT7 expressed in insect cells is membrane-bound and capable of hydrolyzing an extracellular chitin substrate, whereas *in vivo*, this enzyme is also released from the plasma membrane and co-localizes with chitin in the entire procuticle. RNAi of *TcCHT7* reveals that this enzyme is nonessential for any type of molt or degradation of the chitinous matrix in the old cuticle. In contrast, TcCHT7 is required for maintaining the integrity of the cuticle as a compact structure of alternating electron-dense and electron-lucent laminae. There is a reduction in thickness of elytral and leg cuticles after RNAi for *TcCHT7*. TcCHT7 is also required for formation of properly oriented long chitin fibers inside pore canals that are vertically oriented columnar structures, which contribute to the mechanical strength of a light-weight, yet rigid, adult cuticle. The conservation of CHT7-like proteins harboring such a unique domain configuration among many insect and other arthropod species indicates a critical role for the group III class of chitinases in the higher ordered organization of chitin fibers for development of the structural integrity of many invertebrate exoskeletons.

## Author summary

Insect cuticle or exoskeleton is an extracellular matrix consisting of three major morphologically distinct layers, the water-proofing envelope, the protein-rich epicuticle and the chitin/protein-rich procuticle. To accommodate growth, insects must periodically replace their cuticles in a process called “molting or ecdysis”. During each molt cycle a new cuticle is deposited simultaneously with degradation of the inner part of the chitinous procuticle of the old one by molting fluid enzymes including epidermal chitinases. We show that a chitinase, CHT7, from the red flour beetle, *Tribolium castaneum*, belonging to a subfamily (group III) of chitinases that have two catalytic domains, is necessary for organization of chitin-containing structures in nascent cuticle, which contributes to the rigidity of the extracellular matrix. This unexpected function is distinct from that of other groups of epidermal chitinases that catalyze the turnover of chitin in old cuticle during the molting process. Because group III chitinases are highly conserved among insect and other arthropod species, we propose that these enzymes have a novel function in processing nascent chitin chains during cuticle assembly and organization into higher order structures that include horizontally stacked laminae and vertically oriented pore canals of many invertebrate cuticular extracellular matrices.

## Introduction

Insect chitinases (CHTs) belong to family 18 glycosylhydrolases (GH-18 superfamily, EC 3.2.1.14) that hydrolyze chitin by an endo-type cleavage to chitooligosaccharides [1–3]. With the availability of many complete genome sequences, it has been recognized that insect genomes encode a large number of chitinases and chitinase-like proteins. These proteins have been classified into eleven groups based on their sequence similarity, domain organization, tissue specificity of expression and phylogenetic analyses [4, 5]. RNA interference (RNAi) studies have indicated functional specialization among these groups of chitinases. Individual groups have been shown to be required for molting, survival, fecundity, proper assembly of cuticle-associated structures, digestion, immunity, and to act as growth factors and to regulate abdominal contraction and wing expansion [6–13]. The functions of the classic group I chitinases with a single catalytic domain and one chitin-binding domain have received the most attention probably because they are highly abundant in the molting fluid, developmentally regulated by insect hormones, and facilitate ecdysis. When group I chitinases are depleted by RNAi, there is a failure of molting in which the insects die trapped in their exuviae [6, 8, 9, 13], suggesting that group I chitinases catalyze the turnover of old cuticle during the molting process. Group II chitinases have multiple catalytic domains and multiple chitin-binding domains and they also are needed for every molt [6, 8]. Some of these catalytic domains do have chitinolytic activity, whereas the catalytically critical glutamate residue in the conserved region II in one or two *N*-terminal domains is substituted for such that those domains are predicted to be noncatalytic [11, 14, 15]. Group IV chitinases have been shown to be expressed predominantly in gut tissue during larval and/or adult stages and are proposed to be involved in digestion of chitin-containing substrates such as peritrophic matrix lining in the midgut or chitinous food material [7, 11, 16, 17]. Group V chitinase-like proteins have been predicted or shown not to possess chitinolytic activity and are called imaginal disk growth factors. RNAi of some of these genes does lead to molting defects and arrested development [6, 8]. The physiological functions of other groups of chitinases are not as well understood. In the brown planthopper, *Nilaparvata lugens*, RNAi for group VI, VII and VIII chitinases (*NlCht6*, *NlCht8* and *NlCht2*, respectively) appears not to cause any morphological abnormalities [9]. In contrast, knockdown of the group VI and VII chitinase genes (*DmCht6* and *DmCht2*, respectively) in the fruit fly, *Drosophila melanogaster*, caused pupal lethality and adult eclosion defects, and the few adults that did develop failed to expand their wings [8]. In addition, DmCht2 has been shown to affect cuticle thickness and laminar organization of *D*. *melanogaster* larval cuticle [18].

In this study we report about a surprising role for a chitinase belonging to the group III subfamily, TcCHT7, from the red flour beetle, *Tribolium castaneum*, in chitin organization rather than chitin degradation in the extracellular matrix. We show that this enzyme with two catalytic domains when expressed in insect cells is membrane-bound and capable of hydrolyzing an extracellular chitin substrate. Its cellular localization *in vivo* suggests that TcCHT7 is appreciably released into the procuticle (both exocuticle and endocuticle) and is associated with chitin where it acts to process the loosely organized chitin crystallites into a more compact structure of alternating electron-dense and electron-lucent layers. This enzyme is also required for formation of properly aligned long chitin fibers that are needed to orient the vertical pore canals in the exocuticle. We further show that TcCHT7 is important for the proper organization of both hard and soft cuticle at all developmental stages. Even in the adult stage, it plays critical roles in laminar organization of the mesocuticle and in formation of pseudo-orthogonal macrofibers in the endocuticle. We propose that TcCHT7 is required during the early stages of chitin macrofiber assembly, presumably for limited trimming of newly formed chitin nanofibrils into products of a uniform size and organizing them into higher ordered chitin crystallites [19] in both the horizontal laminae and the vertical chitin fibers in the pore canals of the beetle’s extracellular matrix.

## Results

### Group III chitinases share an evolutionarily conserved domain organization

TcCHT7 is predicted to be a membrane-bound protein with an N-terminal transmembrane domain, followed by two tandem catalytic domains and one six-cysteine-containing chitin-binding domain (CBD) related to the peritrophin A domain (CBM14, pfam01607) at the C-terminus (S1A Fig). There is a report suggesting that another group III chitinase, SfCHT7 from the white-backed planthopper, *Sogatella furcifera*, is a nonmembrane-bound secreted protein with a signal peptide of 24 amino acids predicted to occur in its N-terminal region [12]. A group III chitinase from the Asian corn borer, *Ostrinia furnacalis*, OfChtIII, containing a transmembrane signature motif was predicted to be a membrane-bound enzyme [20]. In the genomes of all other insect species (Hexapoda) and several subphyla of Arthropoda in the databases, a single ortholog was identified using TcCHT7 as the query. Phylogenetic analysis of the two catalytic domain sequences of TcCHT7 and of other insect and arthropod orthologs showed that the first catalytic domain of these CHT7s exhibits greater sequence similarity to one another than to catalytic domain 2 in the same protein, suggesting distinct functions and/or evolutionary origins for each of the two catalytic domains (S1B Fig). Note that both catalytic domains contain the four signature motifs (conserved motifs I to IV located in strands β3, β4, β6 and β8) characteristic of the GH-18 superfamily of chitinases (S2 Fig) [11]. Furthermore, the glutamate residue (E) in motif II, DXDW**E**(Y/F)P, which is the most critical residue that acts as the proton donor required for the cleavage of the glycosidic bond [14], is retained in both catalytic domains of all of the CHT7s analyzed (S2 Fig). These results suggest that both catalytic domains are catalytically active. Manifestly, the two catalytic domains of OfChtIII from *O*. *furnacalis* expressed in *Pichia pastoris* were shown to have catalytic activity [20]. The nematode genomes have a chitinase phylogenetically related to insect group III chitinases, but the nematode chitinases have only a single catalytic domain and one or two chitin-binding domains [5]. The biochemistry and developmental role(s) of group III chitinases have not been well studied and our results will help to remedy this deficiency in our understanding of the enzyme’s function(s).

### Recombinant TcCHT7 hydrolyzes chitin present in extracellular space

**Fig 1:**
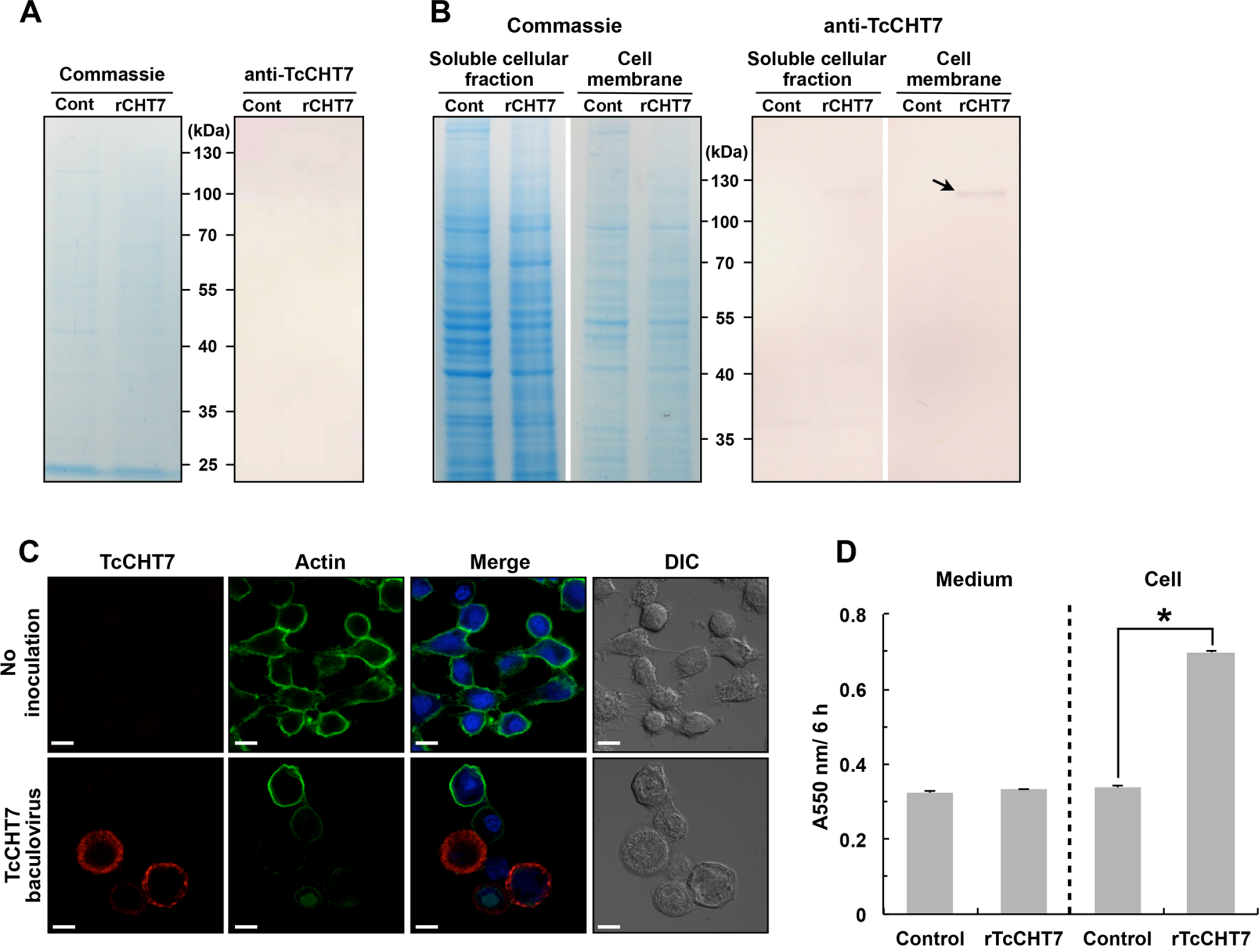
Recombinant TcCHT7 expression in insect cell line. Recombinant virus for expression of recombinant TcCHT7 (rTcCHT7) was constructed using BaculoGold DNA and added to monolayers of Hi-5 cells. All experiments were carried out at 3 days after addition of the *TcCHT7*-baculoviruses. (A and B) SDS-PAGE analysis and immunoblotting of rTcCHT7. Protein samples of the cell culture medium (A) and soluble cellular and cell-membrane fractions (B) were analyzed by 10% SDS-PAGE followed by Coomassie Blue staining or western blotting using the anti-TcCHT7 antibody. An immunoreactive band with the predicted mass of rTcCHT7 (~111 kDa) was detected in the cell-membrane fraction (arrow). (C) Immunocytochemical analysis of rTcCHT7. The *TcCHT7*-baculoviruses-infected Hi-5 cells were incubated with anti-TcCHT7 antibody, which was then detected by Alexa Fluor 546 goat anti-rabbit IgG (red). Actin and nuclei were stained with Alexa Fluor 488 Phalloidin (green) and ToPro-3 (blue), respectively. Scale bar = 10 μm. (D) Chitinolytic activity of rTcCHT7. The cell culture medium was gently replaced with 0.5 mg/ml CM-chitin-RBV dissolved in 0.2 M phosphate buffer, pH 8, and incubated at 27°C for 6 h. The medium was stored at 4°C until enzyme assay. Non-infected Hi-5 cells were used as negative controls. An asterisk indicates a significant difference in chitinolytic activity between test and control cells (p = 6.17E-05, *t*-test). Data are shown as the mean value ± SE (n **=** 3).

Analysis of the amino acid sequence of the TcCHT7 protein by the TMHMM program [21] indicated the presence of a transmembrane segment near the N-terminus and that its catalytic domains are exposed to the cuticle side of the plasma membrane. To confirm these predictions, we expressed this protein in Hi-5 insect cells infected with a recombinant baculovirus encoding the full-length coding sequence of the *TcCHT7* gene. Likewise, Zhu et al. [22] expressed recombinant chitinases and chitinase-like proteins belonging to group I (rTcCHT5, rDmCHT5 and rMsCHT5 from *T*. *castaneum*, *D*. *melanogaster* and the tobacco hornworm, *Manduca sexta*, respectively), group IV (rDmCHT4 and rDmCHT9) and group V (rTcIDGF2, rTcIDGF4 and rDmDS47), all of which contained a predicted signal peptide. All of those proteins were secreted into the cell culture medium. In contrast, a protein of the expected molecular mass of rDmCHT7 (group III), which has a predicted membrane-anchoring segment at the N-terminus, was found in the cell pellet fraction but not in the medium [22]. Similarly, recombinant TcCHT7 (rTcCHT7) protein was not detected in the culture medium by an antibody specific for this protein generated using a synthesized peptide (G^457^-G^474^) as the antigen, which corresponds to the linker region between the two catalytic domains of the TcCHT7 protein (Fig 1A). Instead, an immunoreactive protein (arrow in Fig 1B) with a predicted mass of ~111 kDa for rTcCHT7) was detected in the pellet fraction containing the cell membrane. Furthermore, immunocytochemical analysis shows that at least a part of rTcCHT7 is localized in the cell membrane fraction of the *TcCHT7*-baculovirus-infected Hi-5 cells (Fig 1C), a result consistent with the prediction that this protein has a transmembrane segment at the N-terminus and lacks a cleavable signal peptide.

To further confirm the membrane location and the orientation of the catalytic domains, we measured chitinolytic activity of the culture medium and the washed Hi-5 cell monolayer fraction that was attached intact to the plastic culture flask following infection with a baculovirus expressing *TcCHT7*. Whereas high chitinolytic activity was detected in the cell culture medium from expressed recombinant chitinases belonging to groups I and IV from several insect species [22–24], little or no chitinase activity was observed in the medium from the rTcCHT7 expression relative to the control medium (Fig 1D). Instead, chitinase activity was observed in the assay of the washed cell monolayers when the substrate was added to the medium containing the transformed Hi-5 cells (Fig 1D). These results are consistent with rTcCHT7 being anchored in the cell membrane and with one or both of its catalytic domains facing the extracellular side (equivalent to the cuticle side *in vivo*).

### Expression pattern of *TcCHT7* parallels that of CHS-A

Real-time PCR was performed to determine the expression pattern of *TcCHT7* during development. *TcCHT7* transcripts were detected at all developmental stages analyzed except at the mature adult stage (3–4 weeks-old adults) (S3A Fig). More detailed studies showed that *TcCHT7* transcripts were detected at their highest levels right after pupation and adult eclosion (S3B Fig). Like that seen with *O*. *furnacalis* [20], the temporal expression profile of *TcCHT7* is similar to that of *chitin synthase-A* (*TcCHS-A*) in *T*. *castaneum* (S4 Fig) [25, 26], which catalyzes the synthesis of chitin in newly formed cuticle, but it is distinctly different from that of *TcCHT5* (S4 Fig), which is involved in degradation of chitin in the old cuticle during molting. The transcript level of *TcCHT7* in the elytron dissected from 0 d-old adult was nearly the same as that in the membranous hindwing (left panel in S3C Fig). Similarly, *TcCHT7* transcripts were detected in both the adult ventral abdomen, whose cuticle is highly sclerotized and rigid, and the adult dorsal abdomen, whose cuticle is transparent, flexible and membranous (right panel in S3C Fig). These results indicate that TcCHT7 plays a role in the formation of both rigid and soft adult cuticles rather than in the degradation of chitin in the old pupal cuticle just prior to adult eclosion of *T*. *castaneum*.

### Localization of TcCHT7 protein in the cuticle

**Fig 2:**
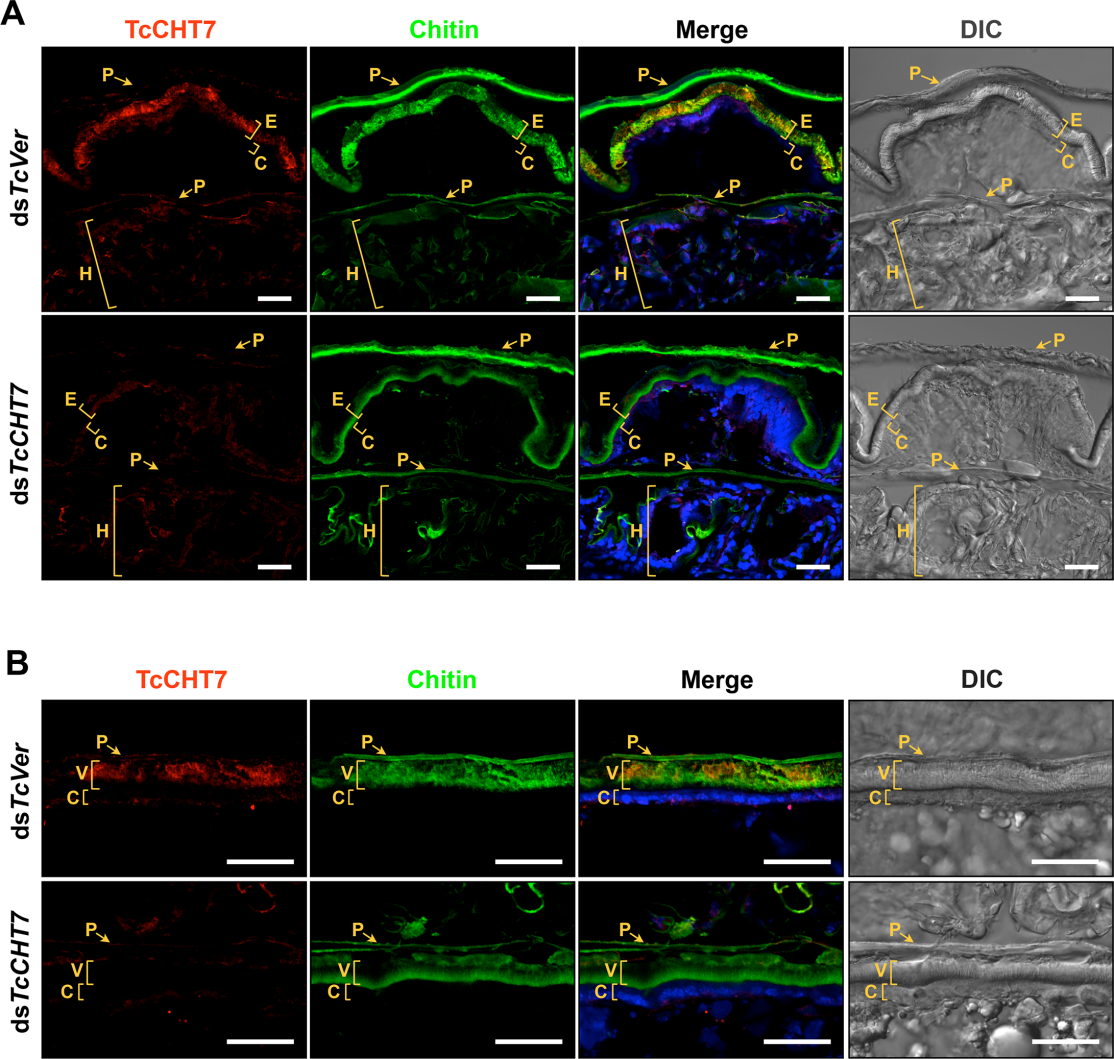
Localization of TcCHT7 protein in adult cuticle. Immunohistochemical analysis was performed to determine the locations of TcCHT7 in adult elytral (A) and ventral abdominal (B) cuticles. Cryosections of pharate adults (5 d-old pupae) that had been injected with ds*TcCHT7* or ds*TcVer* in the late larval stage were incubated with the anti-TcCHT7 antibody, which was then detected by Alexa Fluor 546-conjugated goat anti-rabbit IgG (red). Cuticular chitin and nuclei were stained with FITC-conjugated chitin-binding protein probe (green) and To-Pro-3 (blue), respectively. P, pupal cuticle; E, elytral dorsal cuticle; C, epidermal cell; H, hindwing; V, ventral abdominal cuticle. Scale bar = 20 μm.

An immunohistochemical method was used to localize the TcCHT7 protein in the cuticles of pharate adults (5 d-old pupae) of *T*. *castaneum*. Unlike the OfChtIII protein, which appears to be co-localized with OfCHS-A in the apical epidermal cell plasma membrane but not in the chitinous cuticle in *O*. *furnacalis* on day 1 of the fifth instar [20], TcCHT7 is co-localized with chitin in the rigid elytral dorsal cuticle and abdominal membranous ventral cuticle on day 5 of the pupal stage (Fig 2A and 2B). We do not know if the localization changes during pupal development. To localize the TcCHT7 protein in rigid cuticles more precisely, we further performed transmission electron microscopy and immunolabeling with TcCHT7 antibody and a gold-conjugated secondary antibody. In the elytral dorsal cuticle, TcCHT7 is predominantly detected in both the horizontally oriented laminae and the vertically oriented pore canal fibers (PCFs) throughout the procuticle, as well as in the apical plasma membrane (S5 Fig). This finding is in contrast to the results of the expression of TcCHT7 in recombinant baculovirus-infected insect cells described in Fig 1, suggesting that TcCHT7 is released from the plasma membrane of the underlying epidermal cells *in vivo* during cuticle maturation by an unknown mechanism. TcCHT7 was also detected in membranous hindwing cuticle (Fig 2A), but it appeared to be reduced to a level proportional to the lower chitin content of this tissue. It is noteworthy that TcCHT7 is not detected in the old pupal cuticle, suggesting that this protein turns over as the cuticle matures (Fig 2A). This situation is similar to the finding that Knickkopf (TcKnk), a GPI-anchored membrane protein, is also found throughout the newly forming adult procuticle and undetectable in the old pupal cuticle in *T*. *castaneum* [27].

### dsRNA-mediated loss of function phenotype

**Fig 3:**
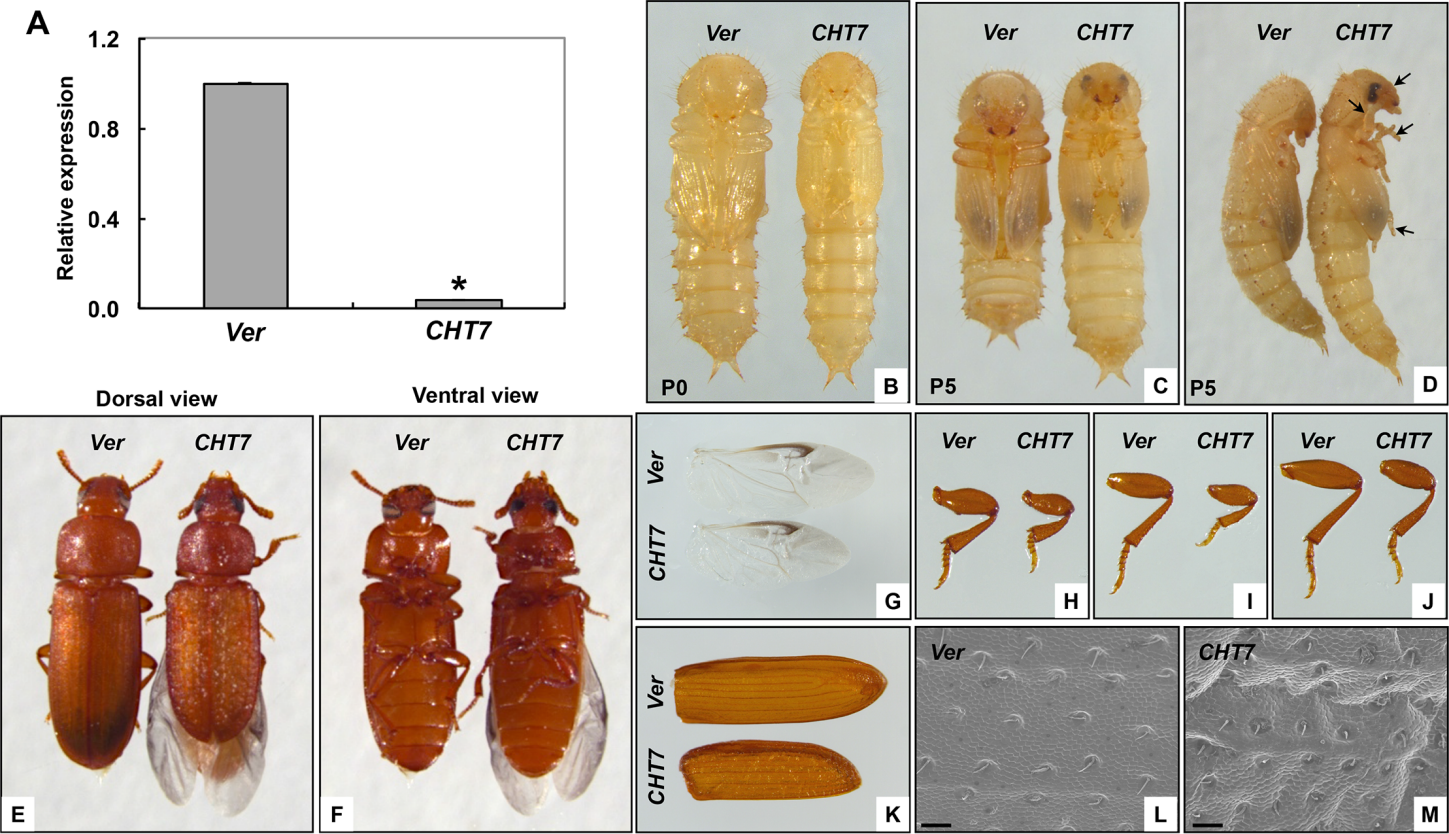
Phenotypes produced by RNAi of *TcCHT7*. ds*TcCHT7* (*CHT7*) or ds*TcVer* (*Ver*) was injected in the late larval stage (200 ng per insect; n = 30). (A) To analyze knockdown level of *TcCHT7* transcripts by real-time PCR, total RNA was isolated from pharate adults (5 d-old pupae). Expression levels of *TcCHT7* in ds*TcCHT7*-treated insects are presented relative to the levels in the ds*TcVer*-treated control. An asterisk indicates a significant difference in transcript levels of *TcCHT7* between control and test insects (p = 4.76E-05, *t*-test). Data are shown as the mean value ± SE (n **=** 3). (B-D) Injection of ds*TcCHT7* had no effect on larval-pupal molting or pupal development. However, the resulting pupae failed to undergo abdominal contraction, wing expansion or tightening of head, antenna and legs (arrows in D). The resulting adults (E and F) exhibited abnormally small appendages including hindwing (G), fore leg (H), middle leg (I), hind leg (J) and elytron (K) compared to those of ds*TcVer*-treated control (See S6 Fig). Morphology of the dorsal elytral cuticle was analyzed by SEM (L and M; scale bar = 20 μm).

To investigate the function of *TcCHT7* in *T*. *castaneum*, we performed double-stranded RNA (dsRNA)-mediated transcript down-regulation (RNA interference, RNAi). RNAi of *TcCHT7* led to a substantial decrease in expression of the *TcCHT7* gene at both the mRNA (Fig 3A) and protein levels (Fig 2). As we had previously reported [6], injection of ds*TcCHT7* into late instar larvae had no effect on larval development or the larval-pupal molt. However, several morphological abnormalities were observed right after pupation. The resulting pupae failed to contract their abdomens (Fig 3B). In addition, other critical events for pupal maturation such as expansion of the forewings and pronotum as well as extension and/or folding of the legs, antennae and head were negatively affected (Fig 3C and 3D).

Unlike *D*. *melanogaster* in which RNAi for *DmCHT7* caused a lethal pupal-adult molting defect [8], the TcCHT7-deficient pupae, nevertheless, developed and did molt into adults (Fig 3E and 3F). However, the resulting adults exhibited significantly smaller appendages including the wings and legs (Figs 3G–3K and S6). Furthermore, the surface of the elytron was rougher and wrinkled, instead of being smooth as in the ds*TcVer*-treated control insects (Fig 3E, 3L and 3M). In addition, the hindwings did not fold properly under the elytra (Fig 3E and 3F). Although RNAi for *TcCHT7* resulted in smaller legs, it affected neither the movement of the leg joints/limbs nor the locomotion of the adult. However, TcCHT7-deficient adults toppled over onto their backs frequently and had difficulty up-righting their bodies compared to control adults (see S1 and S2 Videos). RNAi of *S*. *furcifera SfCHT7* has been shown to result in insects that exhibited difficulty in molting and wing development [12]. A lethal phenotype occurred when planthopper nymphs were able to detach from the exuvia of the head capsule cuticle, but not from the old cuticle of the nymph’s body. Another lethal phenotype consisted of fifth-instar nymphs with elongated distal wing pads and abnormally thin junctions between the thorax and abdomen, resulting in a “wasp-waisted” appearance. In another phenotype that was nonlethal, old cuticles detached completely from the body, but the wings of the adult did not expand normally. These results indicated that SfCHT7 is involved in functions other than molting.

### TcCHT7 is required for laminar organization of the procuticle at all developmental stages

**Fig 4:**
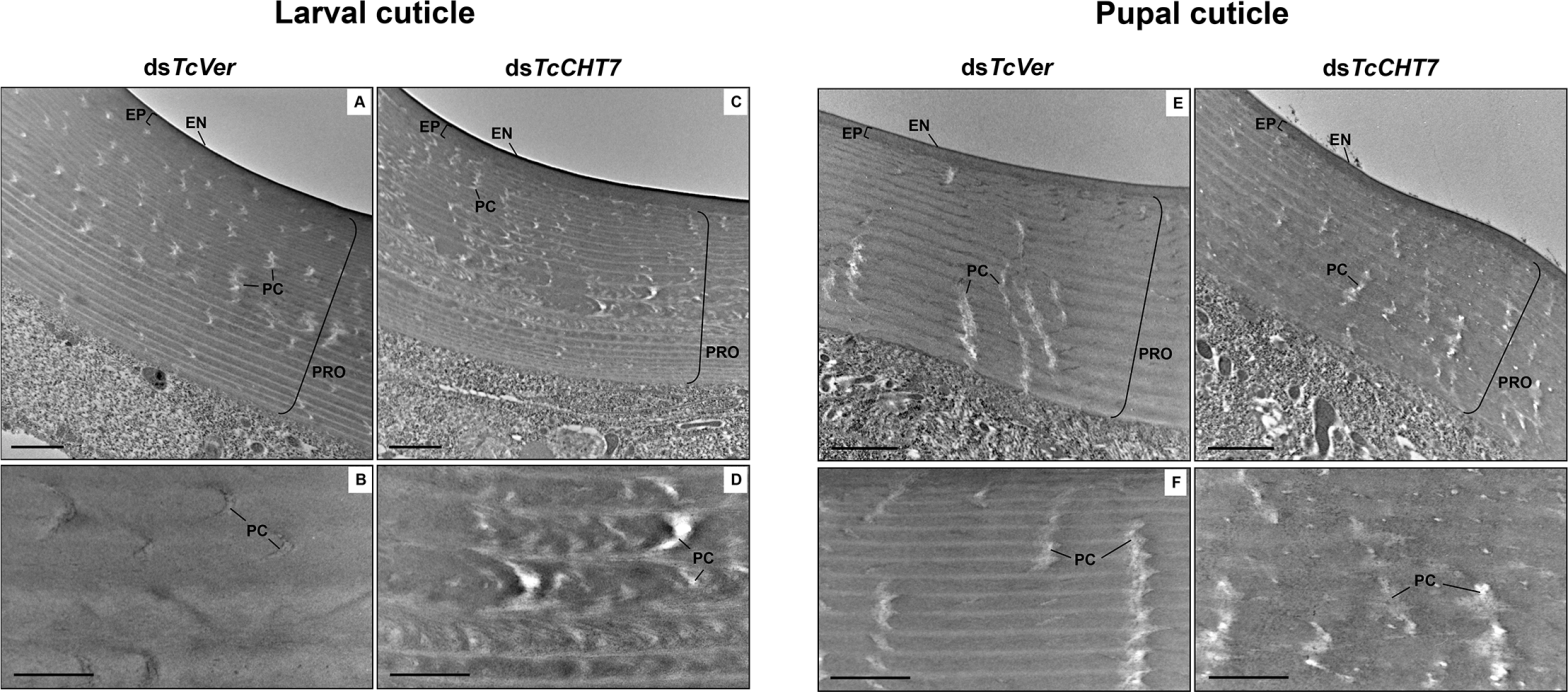
Ultrastructure of larval and pupal cuticles from TcCHT7-deficient insects. ds*TcCHT7* or ds*TcVer* was injected at the young larval stage (200 ng per insect; n = 30). The ultrastructure of larval (A-D) and newly forming pupal (E-H) cuticles from late instar larvae and pharate pupae, respectively, was analyzed by TEM. The middle portions of the larval and pupal cuticles are enlarged in bottom panels. EN, envelope; EP, epicuticle; PRO, procuticle; PC, pore canal. Scale bar in A, C, E and G = 1 μm; B, D, F and H = 500 nm.

Insect cuticle is composed of several morphologically distinct layers such as the envelope, epicuticle and procuticle [28]. The procuticle can be further divided into two layers, exocuticle and endocuticle, both with a variable number of horizontal chitin-protein rich laminae parallel to the apical plasma membrane of the epidermal cells, depending on the stage of development [29, 30, 33]. The effect of RNAi of *TcCHT7* on the ultrastructure of the cuticle was investigated by TEM throughout multiple stages of *T*. *castaneum* development. The larval dorsal body wall and pupal cuticles of ds*TcVer*-treated control insects exhibited well-organized, horizontal alternating electron-lucent and electron-dense laminae, as well as electron-lucent pore canals, which follow a twisted corkscrew- or ribbon-like helicoidal path reaching all the way to the epicuticle as they traverse the parallel stacks of laminae in the procuticle (Fig 4A, 4B, 4E and 4F). Injection of ds*TcCHT7* into penultimate instar larvae did not prevent the subsequent molt, but the newly formed larval body wall cuticle exhibited poorly defined laminae compared to controls and irregular pore canals (Fig 4C and 4D). In addition, the pupal cuticle at the pharate pupal stage continued to have less compact laminae with indistinct boundaries in the procuticle and imperfectly formed pore canals (Fig 4G and 4H). These results indicated that TcCHT7 is critical for proper laminar organization and pore canal formation in the chitinous procuticle of larval and pupal cuticles of *T*. *castaneum*. We did not observe either cuticle detachment or blister-like structures as reported in third instar larvae of *D*. *melanogaster* after *DmCHT7* RNAi and confocal microscopy [8].

### TcCHT7-deficiency also results in abnormal adult cuticle

**Fig 5:**
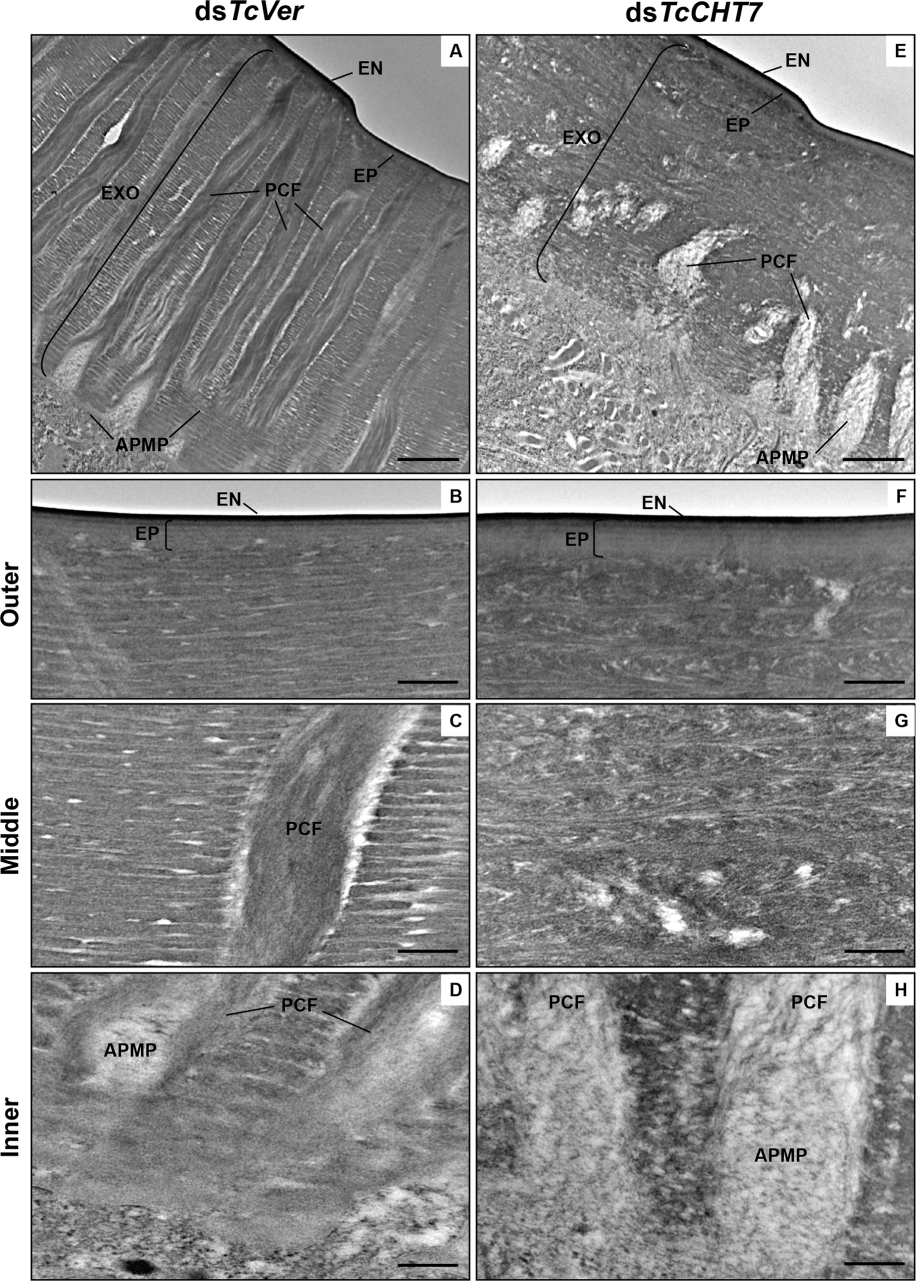
Ultrastructure of elytral dorsal cuticle from TcCHT7-deficient pharate adults. Ultrastructure of rigid elytral dorsal cuticle from pharate adults (5 d-old pupae) that had been injected with dsRNA (200 ng per insect; n = 30) for *TcVer* (A-D) and *TcCHT7* (E-H) into the late instar larvae was analyzed by TEM. The outer (B and F), middle (C and G) and inner (D and H) parts of elytral cuticle are enlarged from A and E. EN, envelope; EP, epicuticle; EXO, exocuticle; PCF, pore canal fiber; APMP, apical plasma membrane protrusion. Scale bar in A and E = 1 μm; B-D and F-H = 200 nm.

The administration of ds*TcCHT7* during larval stages led to small, but significant, reductions in thickness of elytral and leg cuticles compared to control adults (19% and 37%, respectively, S7 Fig). There were also persistent ultrastructural abnormities in the adult cuticle as well. In pharate adults (5 d-old pupae), the thick and hard elytral dorsal cuticle from ds*TcVer*-treated control insects exhibited, in addition to the envelope and epicuticle, an exocuticle which was quite different from those of the larval or pupal cuticles (Fig 5A) [30, 31]. The exocuticle was composed of numerous laminae with alternating electron-dense and electron-lucent horizontal layers, which were not stacked helicoidally as inferred from the nearly vertical paths of numerous pore canals containing perpendicularly oriented chitin fibers. These pore canal fibers (PCF) extend directly from the apical plasma membrane protrusions (APMP) of the underlying epidermal cells all the way to the epicuticle (Fig 5A–5D). In contrast, the ultrastructure of the exocuticle of elytra from ds*TcCHT7*-treated insects was quite abnormal (Fig 5E). The outer and middle region of the exocuticle exhibited unorganized and less compacted laminae that lacked clear-cut boundaries between adjacent laminar layers as seen in the controls. A Bouligand type (parabolic) of laminar structure as well as a peacock feather-like structure were evident, which might be indicative of intermediate structures occurring during the formation of stacked laminae or imperfect assemblies due to a limited availability of TcCHT7 (Fig 5F and 5G and S5 Fig). In addition, the innermost region of the exocuticle became remarkably electron-lucent where amorphous fibrous material was evident (Fig 5E and 5H). Only rudimentary pore canals were discernible, and they lacked long PCFs. Similar severe ultrastructural defects were also observed in other body regions covered with rigid cuticle such as the ventral abdomen and legs (S8 Fig).

As reported in several other beetle species [32, 33], two additional morphologically distinct chitinous layers appear in the elytral procuticle of *T*. *castaneum* after adult eclosion. The one denoted as “mesocuticle” is formed underneath the exocuticle by one day after adult eclosion and the other denoted as “endocuticle” (macrofiber or Balken cuticle) continues to be deposited below the mesocuticle from two days after adult eclosion at a rate of approximately one lamina per day and reaches its maximum size of ~9 laminae by 9–10 days after eclosion [31]. In addition to the procuticle, TcCHT7 is also detected in both the mesocuticle and the forming endocuticle in elytra of 3 d-old adults (S9 Fig).

**Fig 6:**
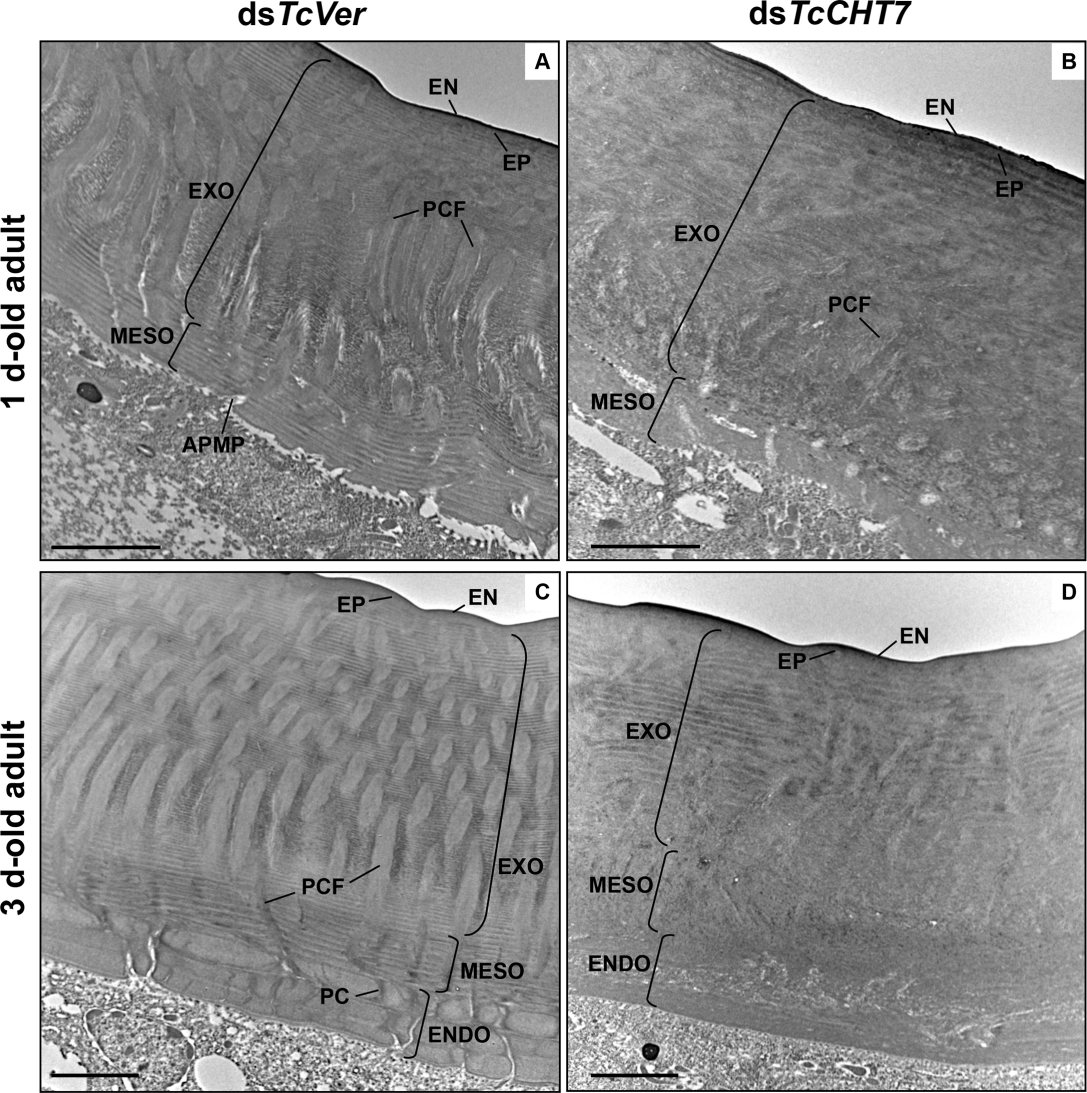
Ultrastructure of mesocuticle and endocuticle from TcCHT7-deficient adults. Ultrastructure of rigid elytral dorsal cuticle from 1 d-old adults (A and B) and 3 d-old adults (C and D) that had been injected with dsRNA (200 ng per insect; n = 30) for *TcVer* (A and C) and *TcCHT7* (B and D) into the late instar larvae was analyzed by TEM. EN, envelope; EP, epicuticle; EXO, exocuticle; PCF, pore canal fiber; APMP, apical plasma membrane protrusion; MESO, mesocuticle; ENDO, endocuticle; PC, pore canal. Scale bar = 2 μm.

To analyze whether a deficiency of TcCHT7 protein affected the ultrastructure of mesocuticle and endocuticle, larvae were treated with ds*TcCHT7* and elytra were dissected from 1 d- and 3 d-old adults. The dorsal elytral cuticle of ds*TcVer*-treated control insects contained normal-looking mesocuticle and the forming endocuticle. The mesocuticle consisted of less compacted horizontal laminae (~10 layers), and the vertical pore canals, which are less prominent than those in the exocuticle, are connected with the wider pore canals in the exocuticle above them (Fig 6A and 6C). The endocuticle is composed of thick brick-like laminae (~3 laminae on day 3 of adult development), which become stacked in an orthogonal or pseudoorthogonal fashion in different beetle species [34] (Fig 6C). In contrast, the mesocuticle and the endocuticle of the elytral dorsal cuticle from ds*TcCHT7*-treated insects at comparable stages lacked the orderly laminar or macrofiber structures and pore canals (Fig 6B and 6D). The exocuticle also had a quite abnormal morphology in which disorganized and amorphous fibrous structures were evident (Fig 6B and 6D). All of these results indicate that TcCHT7 is critical for proper laminar organization and pore canal/PCF formation in the chitinous exocuticle, mesocuticle and endocuticle layers of the rigid extracellular matrix of adult *T*. *castaneum*.

### Formation of soft laminar cuticle is also dependent on TcCHT7

**Fig 7:**
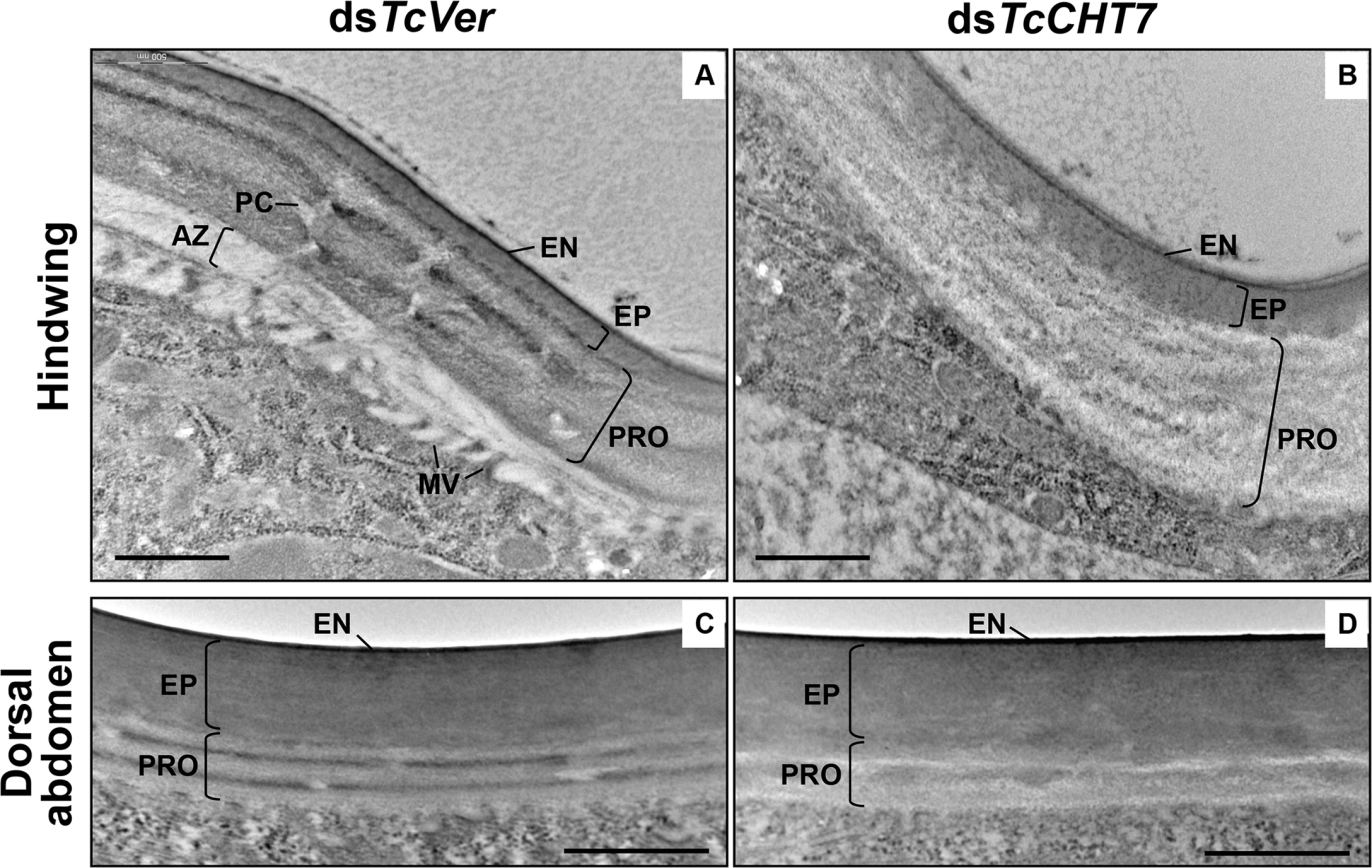
Ultrastructure of hindwing and abdominal dorsal cuticles from TcCHT7-deficient adults. Ultrastructure of membranous and less pigmented hindwing (A and B) and dorsal abdominal (C and D) cuticles from pharate adults (5 d-old pupae) that had been injected with dsRNA (200 ng per insect; n = 30) for *TcVer* (A and C) or *TcCHT7* (B and D) at late instar larval stage was analyzed by TEM. EN, envelope; EP, epicuticle; PRO, procuticle; PC, pore canal; AZ, assembly zone; MV, microvilli. Scale bar = 500 nm.

The membranous hindwing and dorsal abdomen are relatively soft, flexible, thin and less pigmented in *T*. *castaneum* adults than are the forewings and ventral abdomen [30]. Because the *TcCHT7* gene is also expressed in the hindwing and dorsal abdomen (S3C Fig), we analyzed the ultrastructure of these tissues in ds*TcCHT7*-treated insects at the pharate adult (5 d-old pupae) stage. In these soft cuticles from ds*TcVer*-treated control insects, envelope, epicuticle and procuticle layers and plasma membrane-associated microvilli were evident (Fig 7A and 7C). Unlike the rigid adult cuticles such as those of the elytron and ventral abdomen, the procuticle layers from soft cuticles consisted of fewer horizontal laminae and smaller pore canals. Injection of ds*TcCHT7* resulted in abnormal electron-lucent procuticles in the hindwing and dorsal abdomen, exhibiting no distinct electron-dense layers of chitinous horizontal laminae or pore canals. Instead, the procuticle had an amorphous fibrous structure and improperly formed, less electron-dense laminae (Fig 7B and 7D). These results indicate that TcCHT7 plays an important role in an early step in the organization of chitin into laminae and pore canals in soft membranous hindwing and dorsal abdominal cuticles, in addition to its role in organizing more rigid cuticles.

## Discussion

Insect genomes have evolved and expanded to encode a large number of chitinolytic enzymes (>20 in some insect species) that have been grouped into as many as ten unique groups [5]. Insects belonging to different orders vary in the number of groups, but many of them have representatives belonging to groups I through V. RNAi studies have revealed that these groups may serve distinctive functions and expression profiles that are tissue-specific and developmental stage dependent. Even though there are some insect-specific differences, chitinases belonging to each group appear to have conserved functions and domain organizations [6–12].

While several studies have shown that chitinases belonging to multiple groups have degradative functions involving chitin in the extracellular matrices such as cuticle and peritrophic matrix or ingested food material, chitinases belonging to group III have only received limited attention. There is only one member of this group in all insect orders studied. RNAi of group III chitinases results in different phenotypes and lethality in different insect species [6, 8, 9, 12]. In *T*. *castaneum*, RNAi of this gene did not result in mortality during molting but led to shorter wings/elytra and wrinkled cuticles [6]. The results presented here support the hypothesis that TcCHT7 is needed for the maturation and proper organization of the newly formed chitin fibers into cuticular structures (stacked horizontal laminae and vertical pore canals) in the newly forming cuticle of *T*. *castaneum*. This requirement for TcCHT7 applies not only to the thick hardened cuticles such as those found on the elytron and ventral abdomen but also to thinner and membranous cuticles such as those found on the hindwing and dorsal abdomen. This requirement persists in all developmental stages including young adults that are still undergoing cuticle deposition [31]. This is in total contrast to the role of two other chitinases, TcCHT5 and TcCHT10 (belonging to group I and group II, respectively), which are involved exclusively in chitin degradation and the molting of the old cuticle and have more restricted expression profiles [6–10, 13, 20, 27]. The laminar organization of the procuticle and the pore canals is unaffected by RNAi of *TcCHT5* or *TcCHT10* [27]. It has been reported that RNAi of *DmCht2*, a group VII chitinase with a single catalytic domain and no CBD, causes a reduction in cuticle thickness and a loss of laminar organization in *D*. *melanogaster* late third instar larvae [18]. We observed a reduction in thickness of both elytral and leg cuticles after RNAi for *TcCHT7* compared to controls (S7 Fig) and these cuticles had defective organization of horizontal laminae and pore canals, which apparently would affect cuticle rigidity. In view of the finding that TcCHT5 and TcCHT10 are unable to substitute for TcCHT7 in chitin maturation at the pupal/pharate adult stage, the function of TcCHT7 must be different from those of TcCHT5 or TcCHT10. Liu et al. [20] have demonstrated that the two catalytic domains of the group III chitinase from *O*. *furnacalis* hydrolyzed soluble and semi-soluble chitin substrates but not insoluble chitin substrates. Extrapolating this finding to the TcCHT7 enzyme, we propose that a limited trimming rather than extensive degradation of nascent chitin nanofibers during a post-synthetic chitin assembly step is the role performed by TcCHT7. We speculate that the two catalytic domains of the membrane-anchored CHT7 are appropriately positioned to snip the nascent chitin chain as it emerges from the active site of chitin synthase at distinct locations to yield single-stranded chitin fiber precursors. We further propose that several of these chitin chain fragments bound by CHT7 via its C-terminal CBD are released from the epidermal cell membrane and spontaneously associate to form α-chitin crystallites, which are then combined with other chitin-binding proteins [30, 35, 36] to form longer and/or thicker assemblies of chitin fibers in laminar cuticles and pore canals. The kinetics of expression of this protein and its final distribution in the procuticle (horizontal laminae and pore canals) are consistent with this hypothesis. This enzyme has an endo type of action and both catalytic domains are predicted to be catalytically active, presumably during chitin synthesis [20]. The C-terminal CBD and the spacer region between the two tandem catalytic domains found in group III chitinases from all species of arthropods examined may facilitate the binding to nascent chitin and production of cleaved products of a uniform size, which may be essential for the regular higher order organization of chitin nanofibers of the same size. Because TcCHT7 is found both in the laminae and inside the pore canals where laminae are absent, and is present at all developmental stages in epidermal tissues secreting both hard and soft cuticles, TcCHT7 may catalyze a common early step in chitin fiber maturation. This step may involve the formation of α-chitin crystallites and/or elongation of chitin nanofibers, presumably with the assistance of cuticular chitin-binding proteins. The presence of TcCHT7 orthologs with two catalytic domains and a CBD in all subphyla of arthropods analyzed so far, but not in nematodes, indicates that this group of chitinases evolved early during arthropod evolution to accommodate the need for cuticular extracellular matrix morphogenesis [5]. Chitinases belonging to different groups have properties that make them highly attractive for biotechnological applications and show enough diversity to provide species-specific targets for use in pest control [37–39].

## Materials and methods

### Insects

*T*. *castaneum* GA-1 strain [40] was used to study adult cuticle development. Insects were reared at 30°C at 50% relative humidity in whole wheat flour fortified with 5% (v/v) Brewer’s yeast. Under these rearing conditions, adult eclosion occurs 5 days after pupation.

### Phylogenetic analysis

Chitinase-related proteins belonging to group III with two catalytic domains and a peritrophin-A type chitin-binding domain were identified by a BLAST search of the NCBI protein database using the prototypic member of this group from *T*. *castaneum*, TcCHT7 [6] as the query. The CHT7 sequences were aligned using the ClustalW software prior to phylogenetic analysis. The MEGA 7 program [41] was used to construct the phylogenetic tree using the neighbor-joining method. A bootstrap analysis of 5,000 replications was performed to evaluate the branch strength of the phylogenetic tree. The accession numbers of all protein sequences used in this study are shown in S1 Table.

### Expression of recombinant TcCHT7 (rTcCHT7) protein

The full coding sequence including the putative transmembrane segment of *TcCHT7* was amplified from the cloned cDNA (GenBank accession number DQ659247) (Zhu et al., 2008b) by PCR using the primer set: 5’-TGC TCT AGA **ATG** ATA CCG CGA CCC CGT TGC-3’ and 5’-GAA GAT CTT **TAA** TGA TGA TGA TGA TGA TGT CGC TTA GTG GGA GGC GCAG-3’, which contain XbaI and BglII recognition sites (underlined) and translation start and termination codons (bold), respectively. The PCR product was digested with XbaI and BglII, and then subcloned into a similarly digested pVL1393 transfer plasmid DNA (Pharmingen, San Diego, CA). Recombinant baculovirus for expression of the *TcCHT7* gene was constructed using the BD BaculoGold baculovirus expression system (BD Biosciences) and the pVL1393-TcCHT7 vector as described previously [23]. Monolayers of Hi-5 insect cells were inoculated with the recombinant baculovirus and incubated for 3 d at 27°C. The culture medium and cells were collected by centrifugation at 5,000 g for 5 min. The cells were homogenized in 10 mM phosphate buffered saline (pH 7.4) containing a protease inhibitor cocktail (Thermo Scientific), and centrifuged at 13,000 g for 5 min at 4°C. The supernatant was collected as the soluble cellular fraction and boiled in SDS sample buffer. The cell pellet (membrane fraction) was dissolved in SDS sample buffer, heated at 95°C for 10 min and then centrifuged at 13,000 g for 2 min. The supernatant was collected as the detergent-solubilized cell-membrane fraction. Protein samples were analyzed by 10% SDS-PAGE followed by Coomassie Blue staining or western blotting using the anti-TcCHT7 polyclonal antibody generated by immunization of rabbits with a synthesized peptide ^457^GISRGKNAKDVDWQKIAG^474^ (spanning amino acid sequence positions indicated by the two numbers, which corresponds to the linker region between the two catalytic domains of the TcCHT7 protein) (Young In Frontier Co., Ltd., Korea).

### Chitinolytic activity

The enzyme assay for rTcCHT7 was performed by following the hydrolysis of the polymeric substrate, carboxymethyl Remazol Brilliant Violet-Chitin (CM-Chitin-RBV, Loewe Biochemica). The Hi-5 cell culture medium was gently replaced with 0.2 M phosphate buffer (pH 8) containing the substrate (0.5 mg/ml) 3 d after inoculation of the recombinant *TcCHT7*-baculovirus, and incubated at 27°C for 6 h (the medium was stored at 4°C until the end of the enzyme assay). The substrate overlay solution was collected and mixed with an equal volume of 2 N HCl to terminate the reaction. The mixture was placed on ice for 10 min and then centrifuged at 12,000 g for 5 min. The absorbance of the supernatant was measured at 550 nm. For the measurement of chitinolytic activity in the collected culture medium from Hi-5 cells, 150 μl of a reaction mixture consisting of the substrate (0.5 mg/ml) and 50 μl of the medium was incubated at 37°C for 2 h, after which chitinase assay was done in the same way as described above.

### Real-time PCR

To analyze developmental expression profile of *TcCHT7*, total RNA was isolated from whole insects (n = 5–10 except for embryos) at various developmental stages from embryos to adults by using the RNeasy Mini Kit (Qiagen), and then cDNAs were synthesized with an oligo-(dT) primer using the SuperScript III First-strand Synthesis System (Invitrogen) according to manufacturer’s instructions. To analyze tissue-specific expression patterns of *TcCHT7*, elytra, hindwings, ventral and dorsal integuments from 0 d-old adults (n = 10) were dissected to isolate total RNA. Real-time PCR were performed as described previously [30] using the primers 5’-ACG CCA GAC AGA CGT TCA TCT TCA-3’ and 5’-TTT GAT TTC CTG GGC TTC GGC TTC-3’ for *TcCHT7*. The primers 5’-ACG CAA GTC AGT TAG AGG GTG CAT-3’ and 5’-TCC TGT TCG CCT TTA CGC ACG ATA-3’ were used to amplify transcripts for *T*. *castaneum* ribosomal protein S6 (*TcRpS6*) to normalize for differences between the concentrations of cDNA templates. Real-time PCR was performed in a 50 μl reaction volume containing 1 μl of template cDNA, 25 μl SYBR Premix Ex Taq (TAKARA), 0.1 μM of each primer using the Thermal Cycler Dice real-time PCR system (TAKARA). Real-time PCR was carried out with initial denaturation at 95°C for 30 s followed by 40 cycles of 95°C for 5 s and 60°C for 30 s. At the end of the PCR reaction, a melt curve was generated to evaluate the possibility of undesirable side-products.

### RNA interference (RNAi)

Double-stranded RNA (dsRNA) for *TcCHT7* (ds*TcCHT7*, 550 bp) was synthesized as described previously [42] using the primer set 5’-(T7)-TAC GAA ACG TCG ATC-3’ and 5’-(T7)-CGC TAC ATC TAC AAT G-3’. T7 indicates the T7 RNA polymerase recognition sequence (TAA TAC GAC TCA CTA TAG GGT). ds*TcCHT7* (200 ng per insect) was injected into larvae (a mixture of penultimate instar and last instar larvae) (n = 30). dsRNA for the *T*. *castaneum Vermilion* gene (ds*TcVer*) [43] was injected to serve as a negative control. To analyze the levels of *TcCHT7* transcripts after RNAi, total RNA was isolated from whole insects (5 d-old pupae) (n = 3). Total RNA was independently isolated for each of the three replications and significant differences were analyzed using the Student *t*-test.

### Immunohistochemistry

To analyze localization of TcCHT7 protein, cryosections (~12 μm) of pharate adults (5 d-old pupae) treated with ds*TcCHT7* or ds*TcVer* were prepared. Immunohistochemical analysis was carried out as described previously [35]. Sections were washed with PBST (0.01 M phosphate buffered saline, pH 7.4 containing 0.1% Tween 20) three times, and then blocked with blocking buffer (2% bovine serum albumin in PBST) for 1 h at room temperature. The sections were incubated with anti-TcCHT7 antibody (1:50 in blocking buffer) for 3 h at room temperature, washed with PBST three times and then Alexa 546-conjugated goat anti-rabbit IgG (Invitrogen) secondary antibody (1:300 in blocking buffer) was applied for 1 h at room temperature. After washing the sections with PBST, cuticular chitin and nuclei were stained with fluorescein isothiocyanate (FITC)-conjugated chitin-binding protein probe and TO-PRO-3 (Invitrogen), respectively. Tissues were observed using the LSM700 laser scanning confocal microscope (Zeiss) with appropriate filters.

### Electron microscopy

Last instar larvae, pharate pupae (2 d-old prepupae), pharate adults (5 d-old pupae) and 1 d- and 3 d-old adults that had been injected previously with ds*TcCHT7* or ds*TcVer* at the late larval stage of development were collected and fixed in a mixture of 4% paraformaldehyde and 0.1% glutaraldehyde in 0.1 M sodium cacodylate buffer (pH 7.4) for 24 h at room temperature. Ultrastructure of cuticles from the dsRNA-treated animals was analyzed by transmission electron microscopy (TEM) as described previously [30]. For the scanning electron microscopy (SEM), elytra dissected from the dsRNA-treated 3 d-old adults were coated with a gold/palladium mixture and viewed using the S-3500N scanning electron microscope (Hitachi).

## Supporting information

S1 Table Accession numbers of group III chitinases in insects and other arthropods used for phylogenetic analysis. (XLSX)

S1 Video Monitoring of motility of ds*TcVer*-treated control adults. dsRNA (200 ng per insect) for *TcVer* was injected into the late instar larvae, and the locomotion of the resulting adults (1–2 d-old) was monitored using the Stemi 305 stereo microscope (Zeiss) with the MediCAM-Z digital camera (COMART SYSTEM). The videos are played at 5 times faster than normal speed.
(MP4)

S2 Video Monitoring of motility of TcCHT7-deficient adults. dsRNA (200 ng per insect) for *TcCHT7* was injected into the late instar larvae, and the locomotion of the resulting adults (1–2 d-old) was monitored using the Stemi 305 stereo microscope (Zeiss) with the MediCAM-Z digital camera (COMART SYSTEM). The videos are played at 5 times faster than normal speed.
(MP4)

S1 Fig Domain architecture and phylogenetic analysis of insect and other arthropod CHT7s. The SMART program was used to analyze the domains of CHT7s. (A) TcCHT7 contains a single transmembrane span near the N-terminus (gray box), two catalytic domains in the middle and a C-terminal chitin-binding domain (CBD). (B) ClustalW software was used to perform multiple sequence alignments of the catalytic domains identified prior to phylogenetic analysis. The phylogenetic tree was conducted by MEGA7 software using Neighbor-Joining method. Numbers by each branch indicate results of bootstrap analysis of 5,000 replications. See S1 Table for the accession numbers of protein sequences used here.
(TIF)

S2 Fig Alignment of the four conserved motifs in the catalytic domains of group III (CHT7s) insect and other arthropod chitinases. The amino acid sequences of the conserved motifs in the both catalytic domains were aligned using ClustalW. Symbols below the aligned sequences indicate identical (*), highly conserved (:), and conserved residues (.). The glutamate residue (E) in the motif II, which is the most critical residue for chitinolytic activity, is highlighted in red. Group I chitinase (TcasCHT5) from *T*. *castaneum*, which has a single catalytic domain [22], is also included in this analysis (top sequence). See S1 Table for the accession numbers of protein sequences used here.
(TIF)

S3 Fig Expression patterns of *TcCHT7* gene. (A) For the expression profiles of *TcCHT7* by real-time PCR, total RNA was extracted from whole insects at various developmental stages from embryo to adults. E, embryos; YL, young larvae; OL, old larvae; PP, pharate pupae; P, pupae; A, 3–4 w-old adults. (B) To analyze the expression patterns of *TcCHT7* at later stages of development, the time points analyzed between the early pharate pupal and young adult stages were expanded. PP1, 0–1 d-old pharate pupae; PP2, 1**–**2 d-old pharate pupae; P0, 0 d-old pupae; P1, 1 d-old pupae; P2, 2 d-old pupae; P3, 3 d-old pupae; P4, 4 d-old pupae; P5, 5 d-old pupae; A0, 0 d-old adults; A1, 1 d-old adults; A7, 7 d-old adults. Expression levels for *TcCHT7* are presented relative to the levels of expression at the earliest developmental stage analyzed (E or PP1). (C) To analyze the transcript levels of *TcCHT7* in elytra and hindwings (left panel) as well as in ventral and dorsal abdominal cuticle (right panel), total RNA was extracted from tissues of 0 d-old adults. Expression levels for *TcCHT7* are presented relative to the levels of expression in the elytron or ventral abdomen. E, elytron; H, hindwing; VA, ventral abdominal cuticle; DA, dorsal abdominal cuticle. All data are shown as the mean value ± SE (n **=** 3).
(TIF)

S4 Fig Expression patterns of *TcCHS-A* and *TcCHT5* genes. For the expression profiles of *TcCHS-A* and *TcCHT5* by real-time PCR during late developmental stages, total RNA was extracted from whole insects between the early pharate pupal and young adult stages. PP1, 0–1 d-old pharate pupae; PP2, 1**–**2 d-old pharate pupae; P0, 0 d-old pupae; P1, 1 d-old pupae; P2, 2 d-old pupae; P3, 3 d-old pupae; P4, 4 d-old pupae; P5, 5 d-old pupae; A0, 0 d-old adults; A1, 1 d-old adults; A7, 7 d-old adults. Expression levels for *TcCHS-A* and *TcCHT5* are presented relative to the levels of expression at the earliest developmental stage analyzed (PP1). See the legend of S3 Fig for details of expression analysis of *TcCHT7* gene. All data are shown as the mean value ± SE (n **=** 3).
(TIF)

S5 Fig Localization of TcCHT7 protein in rigid elytral dorsal cuticle. Ultra-thin sections of pharate adults (5 d-old pupae) that had been injected with dsRNA (200 ng per insect) for *TcVer* (left panels) and *TcCHT7* (right panels) into the late instar larvae were incubated with anti-TcCHT7 antibody and washed extensively to remove unbound antibody. The antibody was then detected by goat anti-rabbit IgG conjugated to 10 nm gold particles. TcCHT7 protein is present in both horizontally oriented laminae and in vertical pore canal with pore canal fibers (PCF) in their core of the entire procuticle (exocuticle), but not in the envelope and epicuticle layers. The number of gold particles is obviously decreased in TcCHT7-deficient insects. EN, envelope; EP, epicuticle; MV, microvilli; APMP, apical plasma membrane protrusion. Scale bar = 500 nm.
(TIF)

S6 Fig Length of appendages from TcCHT7-deficient insects. Elytra, hindwings and legs were dissected from 3–4 days old adults that had been injected with dsRNA for *TcCHT7* (*CHT7*) and *TcVer* (*Ver*) in the late instar larvae. The length of the elytron (A), hindwing (B), fore leg (C), middle leg (D) and hind leg (E) were measured using *ImageJ* software. An asterisk indicates a significant difference in length between control and test insects (*p* < 0.0001, *t*-test). Data are shown as mean ± SE (n = 6–7).
(TIF)

S7 Fig Thickness of elytral dorsal and leg cuticles from TcCHT7-deficient pharate adults. Ultrastructure of the elytral dorsal and leg cuticles from pharate adults (5 d-old pupae) that had been injected with dsRNA (200 ng per insect) for *TcCHT7* (*CHT7*) and *TcVer* (*Ver*) at the late instar larval stage was analyzed by TEM followed by measurement of thickness of the cuticles. An asterisk indicates a significant difference in thickness between control and test insects (*p* < 0.03, *t*-test). Data are shown as mean ± SE (n = 6).
(TIF)

S8 Fig Ultrastructure of leg and ventral abdominal cuticles from TcCHT7-deficient adults. Ultrastructure of the rigid leg and ventral abdominal cuticles from pharate adults (5 d-old pupae) that had been injected with dsRNA (200 ng per insect) for *TcVer* and *TcCHT7* at the late instar larval stage was analyzed by TEM. EN, envelope; EP, epicuticle; EXO, exocuticle; PCF, pore canal fiber; APMP, apical plasma membrane protrusion. Scale bar = 2 μm.
(TIF)

S9 Fig Localization of TcCHT7 protein in mesocuticle and endocuticle in rigid elytral dorsal cuticle. Ultra-thin sections of 3 d-old adults that had been previously injected with dsRNA (200 ng per insect) for *TcVer* (left panels) or *TcCHT7* (right panels) at the late instar larvae were incubated with anti-TcCHT7 antibody, which was then detected by goat anti-rabbit IgG conjugated to 10 nm gold particles. TcCHT7 protein is present in the exocuticle (middle panels) as well as mesocuticle (MESO) and the endocuticle (ENDO) (bottom panels). The number of gold particles is obviously decreased in TcCHT7-deficient insects. EN, envelope; EP, epicuticle; PCF, pore canal fiber; PC, pore canal.
(TIF)

## References

[1] Henrissat B, Davies G. Structural and sequence-based classification of glycoside hydrolases. Curr Opin Struct Biol. 1997;7(5): 637–44. 9345621 10.1016/s0959-440x(97)80072-3

[2] Lombard V, Golaconda Ramulu H, Drula E, Coutinho PM, Henrissat B. The carbohydrate-active enzymes database (CAZy) in 2013. Nucleic Acids Res. 2014;42: D490–5. doi: 10.1093/nar/gkt1178 24270786 10.1093/nar/gkt1178 PMC3965031

[3] Schomburg I, Chang A, Placzek S, Sohngen C, Rother M, Lang M, et al BRENDA in 2013: integrated reactions, kinetic data, enzyme function data, improved disease classification: new options and contents in BRENDA. Nucleic Acids Res. 2013;41: D764–72. doi: 10.1093/nar/gks1049 23203881 10.1093/nar/gks1049 PMC3531171

[4] Muthukrishnan S, Merzendorfer H, Arakane Y, Yang Q. Chitin metabolic pathways in insects and their regulation In: Cohen E, Moussian B, editors. Extracellular composite matrices in arthropods. Switzerland: Springer; 2016 pp. 31–65.

[5] Tetreau G, Cao X, Chen YR, Muthukrishnan S, Jiang H, Blissard GW, et al Overview of chitin metabolism enzymes in *Manduca sexta*: Identification, domain organization, phylogenetic analysis and gene expression. Insect Biochem Mol Biol. 2015;62: 114–26. doi: 10.1016/j.ibmb.2015.01.006 25616108 10.1016/j.ibmb.2015.01.006

[6] Zhu Q, Arakane Y, Beeman RW, Kramer KJ, Muthukrishnan S. Functional specialization among insect chitinase family genes revealed by RNA interference. Proc Natl Acad Sci U S A. 2008;105(18): 6650–5. doi: 10.1073/pnas.0800739105 18436642 10.1073/pnas.0800739105 PMC2373347

[7] Khajuria C, Buschman LL, Chen MS, Muthukrishnan S, Zhu KY. A gut-specific chitinase gene essential for regulation of chitin content of peritrophic matrix and growth of *Ostrinia nubilalis* larvae. Insect Biochem Mol Biol. 2010;40(8): 621–9. doi: 10.1016/j.ibmb.2010.06.003 20542114 10.1016/j.ibmb.2010.06.003

[8] Pesch YY, Riedel D, Patil KR, Loch G, Behr M. Chitinases and Imaginal disc growth factors organize the extracellular matrix formation at barrier tissues in insects. Sci Rep. 2016;6: 18340 doi: 10.1038/srep18340 26838602 10.1038/srep18340 PMC4738247

[9] Xi Y, Pan PL, Ye YX, Yu B, Xu HJ, Zhang CX. Chitinase-like gene family in the brown planthopper, *Nilaparvata lugens*. Insect Mol Biol. 2015;24(1): 29–40. doi: 10.1111/imb.12133 25224926 10.1111/imb.12133

[10] Zhang D, Chen J, Yao Q, Pan Z, Chen J, Zhang W. Functional analysis of two chitinase genes during the pupation and eclosion stages of the beet armyworm *Spodoptera exigua* by RNA interference. Arch Insect Biochem Physiol. 2012;79(4–5): 220–34. doi: 10.1002/arch.21018 22460420 10.1002/arch.21018

[11] Arakane Y, Muthukrishnan S. Insect chitinase and chitinase-like proteins. Cell Mol Life Sci. 2010;67(2): 201–16. doi: 10.1007/s00018-009-0161-9 19816755 10.1007/s00018-009-0161-9 PMC11115512

[12] Chen C, Yang H, Tang B, Yang WJ, Jin DC. Identification and functional analysis of chitinase 7 gene in white-backed planthopper, *Sogatella furcifera*. Comp Biochem Physiol B Biochem Mol Biol. 2017;208–209: 19–28. doi: 10.1016/j.cbpb.2017.03.002 28363844 10.1016/j.cbpb.2017.03.002

[13] Li D, Zhang J, Wang Y, Liu X, Ma E, Sun Y, et al Two chitinase 5 genes from *Locusta migratoria*: molecular characteristics and functional differentiation. Insect Biochem Mol Biol. 2015;58: 46–54. doi: 10.1016/j.ibmb.2015.01.004 25623241 10.1016/j.ibmb.2015.01.004

[14] Lu Y, Zen KC, Muthukrishnan S, Kramer KJ. Site-directed mutagenesis and functional analysis of active site acidic amino acid residues D142, D144 and E146 in *Manduca sexta* (tobacco hornworm) chitinase. Insect Biochem Mol Biol. 2002;32(11): 1369–82. 12530205 10.1016/s0965-1748(02)00057-7

[15] Chen W, Qu M, Zhou Y, Yang Q. Structural analysis of group II chitinase (ChtII) catalysis completes the puzzle of chitin hydrolysis in insects. J Biol Chem. 2018. 10.1074/jbc.RA117.000119 PMC5827449 29317504

[16] Zhang J, Zhang X, Arakane Y, Muthukrishnan S, Kramer KJ, Ma E, et al Comparative genomic analysis of chitinase and chitinase-like genes in the African malaria mosquito (Anopheles gambiae). PloS one. 2011;6(5): e19899 doi: 10.1371/journal.pone.0019899 21611131 10.1371/journal.pone.0019899 PMC3097210

[17] Shen Z, Jacobs-Lorena M. Characterization of a novel gut-specific chitinase gene from the human malaria vector Anopheles gambiae. J Biol Chem. 1997;272(46): 28895–900. 9360958 10.1074/jbc.272.46.28895

[18] Pesch YY, Riedel D, Behr M. Drosophila Chitinase 2 is expressed in chitin producing organs for cuticle formation. Arthropod Struct Dev. 2017;46(1): 4–12. doi: 10.1016/j.asd.2016.11.002 27832982 10.1016/j.asd.2016.11.002

[19] Neville AC, Parry DA, Woodhead-Galloway J. The chitin crystallite in arthropod cuticle. J Cell Sci. 1976;21(1): 73–82. 932111 10.1242/jcs.21.1.73

[20] Liu T, Zhu W, Wang J, Zhou Y, Duan Y, Qu M, et al The deduced role of a chitinase containing two nonsynergistic catalytic domains. Acta crystallographica Section D, Structural biology. 2018;74(Pt 1): 30–40. doi: 10.1107/S2059798317018289 29372897 10.1107/S2059798317018289 PMC5786006

[21] Krogh A, Larsson B, von Heijne G, Sonnhammer EL. Predicting transmembrane protein topology with a hidden Markov model: application to complete genomes. J Mol Biol. 2001;305(3): 567–80. doi: 10.1006/jmbi.2000.4315 11152613 10.1006/jmbi.2000.4315

[22] Zhu Q, Arakane Y, Beeman RW, Kramer KJ, Muthukrishnan S. Characterization of recombinant chitinase-like proteins of *Drosophila melanogaster* and *Tribolium castaneum*. Insect Biochem Mol Biol. 2008;38(4): 467–77. doi: 10.1016/j.ibmb.2007.06.011 18342251 10.1016/j.ibmb.2007.06.011

[23] Gopalakrishnan B, Muthukrishnan S, Kramer KJ. Baculovirus-Mediated Expression of a *Manduca sexta* Chitinase Gene—Properties of the Recombinant Protein. Insect Biochem Mol Biol. 1995;25(2): 255–65.

[24] Arakane Y, Zhu Q, Matsumiya M, Muthukrishnan S, Kramer KJ. Properties of catalytic, linker and chitin-binding domains of insect chitinase. Insect Biochem Mol Biol. 2003;33(6): 631–48. 12770581 10.1016/s0965-1748(03)00049-3

[25] Arakane Y, Hogenkamp DG, Zhu YC, Kramer KJ, Specht CA, Beeman RW, et al Characterization of two chitin synthase genes of the red flour beetle, *Tribolium castaneum*, and alternate exon usage in one of the genes during development. Insect Biochem Mol Biol. 2004;34(3): 291–304. doi: 10.1016/j.ibmb.2003.11.004 14871625 10.1016/j.ibmb.2003.11.004

[26] Arakane Y, Specht CA, Kramer KJ, Muthukrishnan S, Beeman RW. Chitin synthases are required for survival, fecundity and egg hatch in the red flour beetle, T*ribolium castaneum*. Insect Biochem Mol Biol. 2008;38(10): 959–62. doi: 10.1016/j.ibmb.2008.07.006 18718535 10.1016/j.ibmb.2008.07.006

[27] Chaudhari SS, Arakane Y, Specht CA, Moussian B, Boyle DL, Park Y, et al Knickkopf protein protects and organizes chitin in the newly synthesized insect exoskeleton. Proc Natl Acad Sci U S A. 2011;108(41): 17028–33. doi: 10.1073/pnas.1112288108 21930896 10.1073/pnas.1112288108 PMC3193238

[28] Locke M. The Wigglesworth lecture: Insects for studying fundamental problems in biology. J Insect Physiol. 2001;47(4–5): 495–507. 11166314 10.1016/s0022-1910(00)00123-2

[29] Moussian B, Seifarth C, Muller U, Berger J, Schwarz H. Cuticle differentiation during *Drosophila* embryogenesis. Arthropod Struct Dev. 2006;35(3): 137–52. doi: 10.1016/j.asd.2006.05.003 18089066 10.1016/j.asd.2006.05.003

[30] Noh MY, Kramer KJ, Muthukrishnan S, Kanost MR, Beeman RW, Arakane Y. Two major cuticular proteins are required for assembly of horizontal laminae and vertical pore canals in rigid cuticle of *Tribolium castaneum*. Insect Biochem Mol Biol. 2014;53: 22–9. doi: 10.1016/j.ibmb.2014.07.005 25042128 10.1016/j.ibmb.2014.07.005

[31] Noh MY, Muthukrishnan S, Kramer KJ, Arakane Y. Development and ultrastructure of the rigid dorsal and flexible ventral cuticles of the elytron of the red flour beetle, *Tribolium castaneum*. Insect Biochem Mol Biol. 2017;91: 21–33. doi: 10.1016/j.ibmb.2017.11.003 29117500 10.1016/j.ibmb.2017.11.003

[32] Cheng L, Wang LY, Karlsson AM. Mechanics-based analysis of selected features of the exoskeletal microstructure of *Popillia japonica*. J Mater Res. 2009;24(11): 3253–67.

[33] Leopold RA, Newman SM, Helgeson G. A comparison of cuticle deposition during the pre- and posteclosion stages of the adult weevil, *Anthonomus grandis* Boheman (Coleoptera: Curculionidae). Int J Insect Morphol Embryol 1992;21(1): 37–62.

[34] van de Kamp T, Riedel A, Greven H. Micromorphology of the elytral cuticle of beetles, with an emphasis on weevils (Coleoptera: Curculionoidea). Arthropod Struct Dev. 2016;45(1): 14–22. doi: 10.1016/j.asd.2015.10.002 26529582 10.1016/j.asd.2015.10.002

[35] Noh MY, Muthukrishnan S, Kramer KJ, Arakane Y. *Tribolium castaneum* RR-1 cuticular protein TcCPR4 is required for formation of pore canals in rigid cuticle. PLoS Genet. 2015;11(2): e1004963 doi: 10.1371/journal.pgen.1004963 25664770 10.1371/journal.pgen.1004963 PMC4335487

[36] Vannini L, Willis JH. Localization of RR-1 and RR-2 cuticular proteins within the cuticle of *Anopheles gambiae*. Arthropod Struct Dev. 2017;46(1): 13–29. doi: 10.1016/j.asd.2016.10.002 27717796 10.1016/j.asd.2016.10.002 PMC5292290

[37] Kramer KJ, Muthukrishnan S, Johnson L, White F. Chitinases for insect control In: Carozzi N., K M., editors. Advances in Insect Control: The Role of Transgenic Plants London: Taylor and Francis; 1997 pp. 211–20.

[38] Muthukrishnan S, Merzendorfer H, Arakane Y, Kramer KJ. Chitin metabolism in insects, Gilbert L.I. (Ed.), Insect Molecular Biology and Biochemistry. Gilbert LI, editor. San Diego, pp. 193–235: Elsevier Inc.; 2012.

[39] Merzendorfer H. Insect-Derived Chitinases In: V A., editor. Yellow Biotechnology II Advances in Biochemical Engineering/Biotechnology. 136. Berlin: Springer; 2013 pp. 19–50. 10.1007/10_2013_207 23748348

[40] Haliscak JP, Beeman RW. Status of malathion resistance in five genera of beetles infesting farm-stored corn, wheat, and oats in the United States. J Econ Entomol. 1983;76: 717–22.

[41] Kumar S, Stecher G, Tamura K. MEGA7: Molecular Evolutionary Genetics Analysis Version 7.0 for Bigger Datasets. Mol Biol Evol. 2016;33(7): 1870–4. doi: 10.1093/molbev/msw054 27004904 10.1093/molbev/msw054 PMC8210823

[42] Arakane Y, Muthukrishnan S, Beeman RW, Kanost MR, Kramer KJ. *Laccase 2* is the phenoloxidase gene required for beetle cuticle tanning. Proc Natl Acad Sci U S A. 2005;102(32): 11337–42. doi: 10.1073/pnas.0504982102 16076951 10.1073/pnas.0504982102 PMC1183588

[43] Arakane Y, Lomakin J, Beeman RW, Muthukrishnan S, Gehrke SH, Kanost MR, et al Molecular and functional analyses of amino acid decarboxylases involved in cuticle tanning in *Tribolium castaneum*. J Biol Chem. 2009;284(24): 16584–94. doi: 10.1074/jbc.M901629200 19366687 10.1074/jbc.M901629200 PMC2713571

