# Supplementary figures and images for: A chitinase with two catalytic domains is required for organization of the cuticular extracellular matrix of a beetle
Source: PLoS Genet. 2018 Mar 28;14(3):e1007307. doi: 10.1371/journal.pgen.1007307 (PMC5891080; doi:10.1371/journal.pgen.1007307)

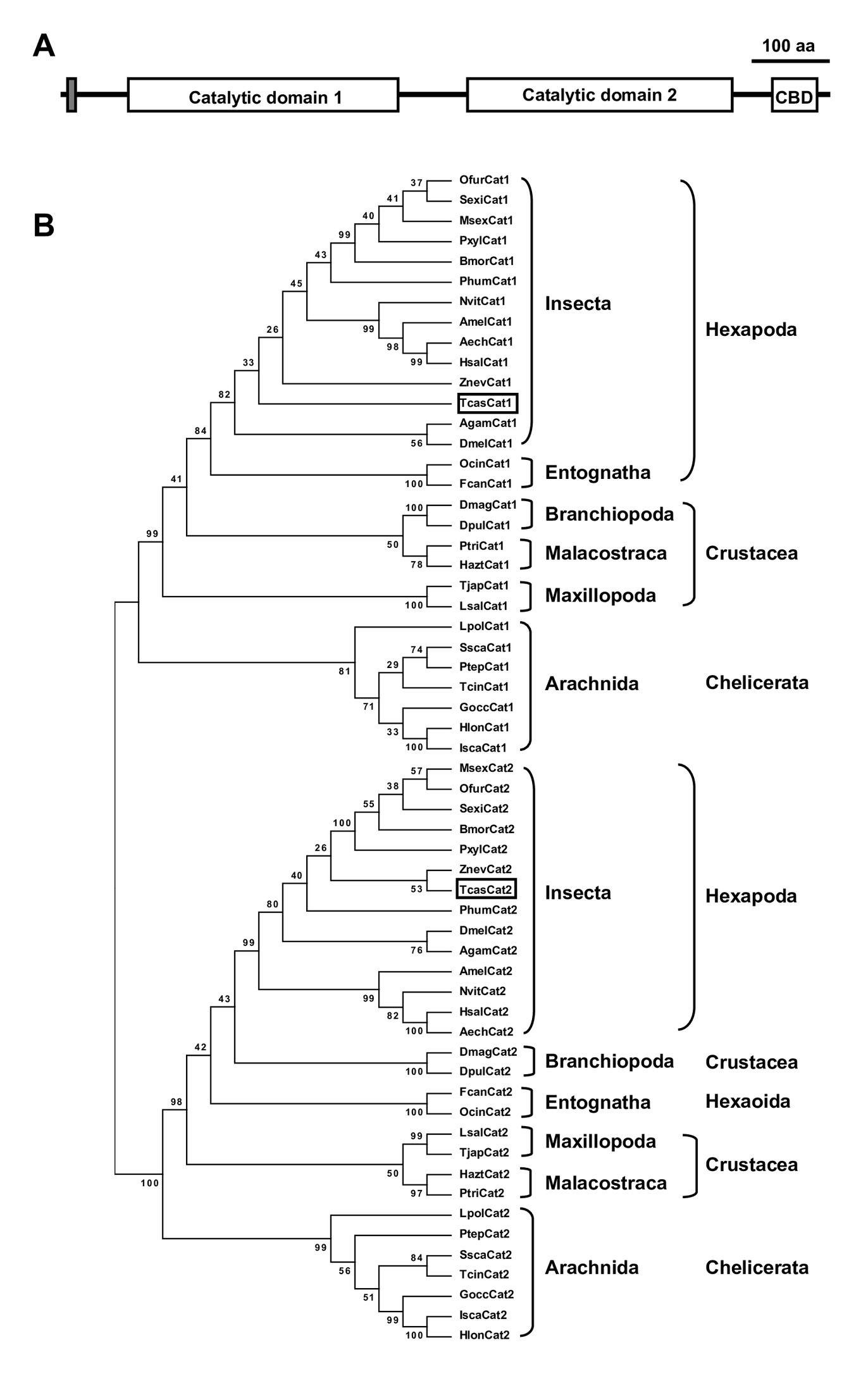

Supplement: S1 Fig — The SMART program was used to analyze the domains of CHT7s. (A) TcCHT7 contains a single transmembrane span near the N-terminus (gray box), two catalytic domains in the middle and a C-terminal chitin-binding domain (CBD). (B) ClustalW software was used to perform multiple sequence alignments of the catalytic domains identified prior to phylogenetic analysis. The phylogenetic tree was conducted by MEGA7 software using Neighbor-Joining method. Numbers by each branch indicate results of bootstrap analysis of 5,000 replications. See S1 Table for the accession numbers of protein sequences used here. (TIF) [file pgen.1007307.s004.tif]

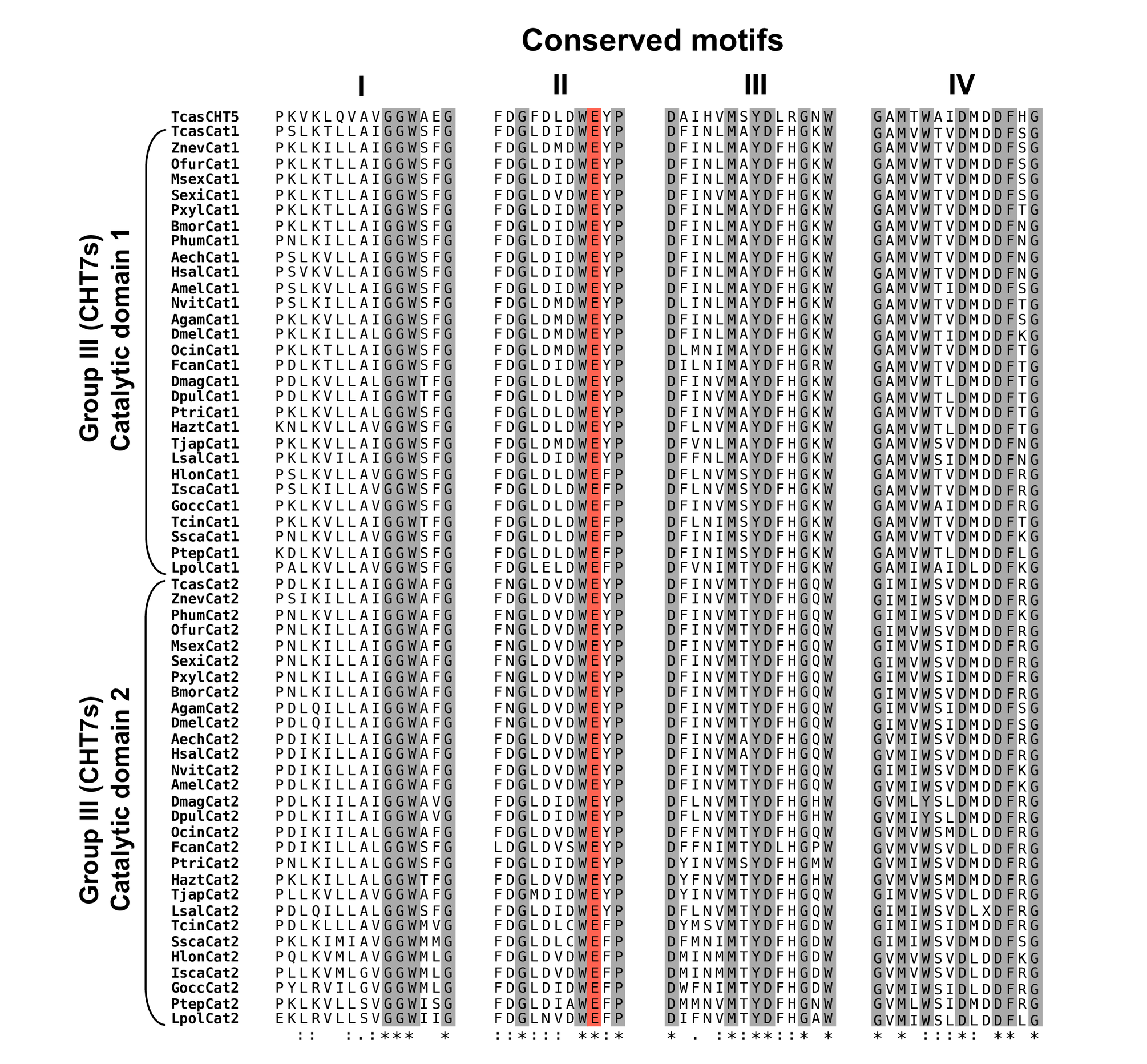

Supplement: S2 Fig — The amino acid sequences of the conserved motifs in the both catalytic domains were aligned using ClustalW. Symbols below the aligned sequences indicate identical (*), highly conserved (:), and conserved residues (.). The glutamate residue (E) in the motif II, which is the most critical residue for chitinolytic activity, is highlighted in red. Group I chitinase (TcasCHT5) from T. castaneum, which has a single catalytic domain [22], is also included in this analysis (top sequence). See S1 Table for the accession numbers of protein sequences used here. (TIF) [file pgen.1007307.s005.tif]

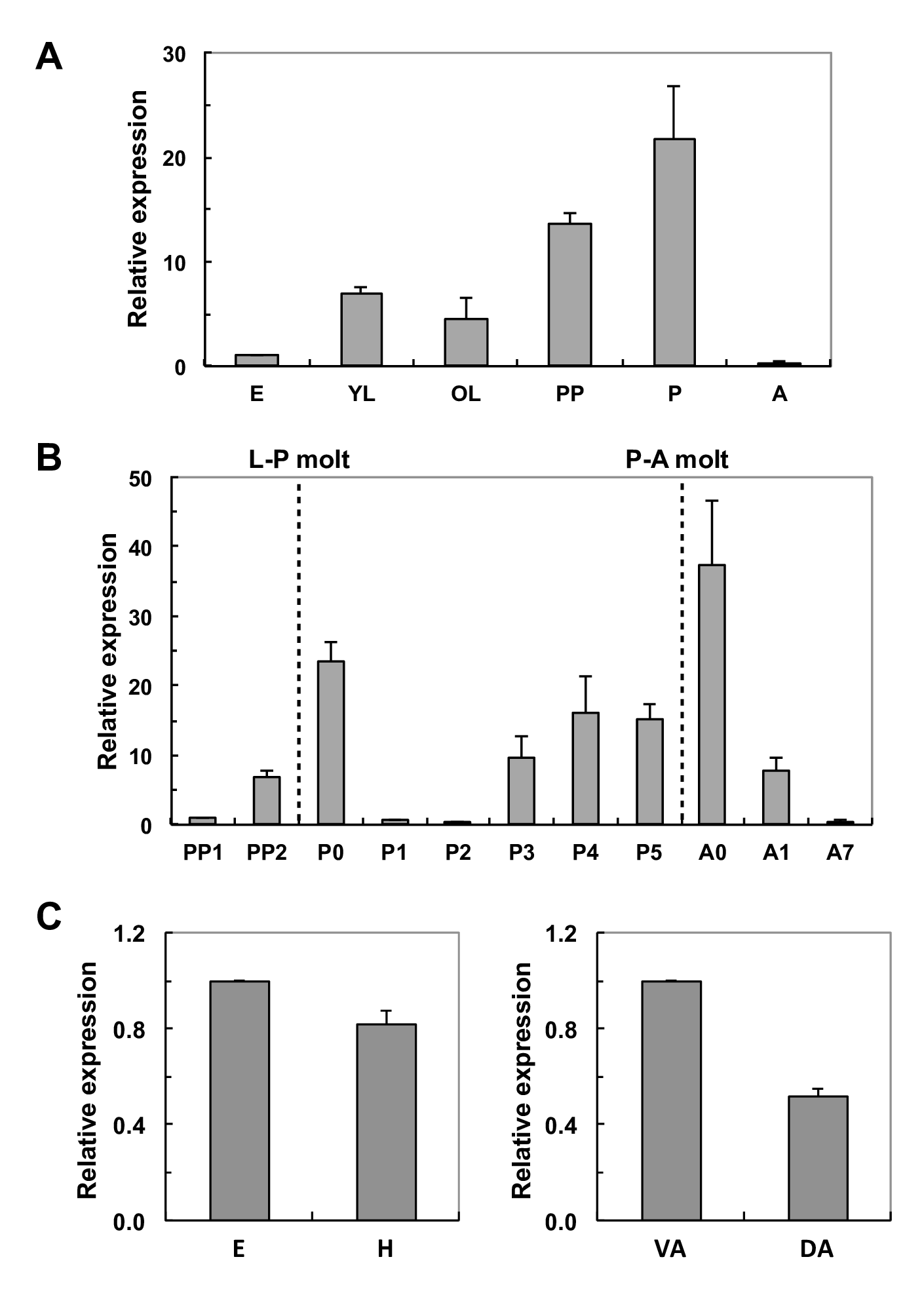

Supplement: S3 Fig — (A) For the expression profiles of TcCHT7 by real-time PCR, total RNA was extracted from whole insects at various developmental stages from embryo to adults. E, embryos; YL, young larvae; OL, old larvae; PP, pharate pupae; P, pupae; A, 3–4 w-old adults. (B) To analyze the expression patterns of TcCHT7 at later stages of development, the time points analyzed between the early pharate pupal and young adult stages were expanded. PP1, 0–1 d-old pharate pupae; PP2, 1–2 d-old pharate pupae; P0, 0 d-old pupae; P1, 1 d-old pupae; P2, 2 d-old pupae; P3, 3 d-old pupae; P4, 4 d-old pupae; P5, 5 d-old pupae; A0, 0 d-old adults; A1, 1 d-old adults; A7, 7 d-old adults. Expression levels for TcCHT7 are presented relative to the levels of expression at the earliest developmental stage analyzed (E or PP1). (C) To analyze the transcript levels of TcCHT7 in elytra and hindwings (left panel) as well as in ventral and dorsal abdominal cuticle (right panel), total RNA was extracted from tissues of 0 d-old adults. Expression levels for TcCHT7 are presented relative to the levels of expression in the elytron or ventral abdomen. E, elytron; H, hindwing; VA, ventral abdominal cuticle; DA, dorsal abdominal cuticle. All data are shown as the mean value ± SE (n = 3). (TIF) [file pgen.1007307.s006.tif]

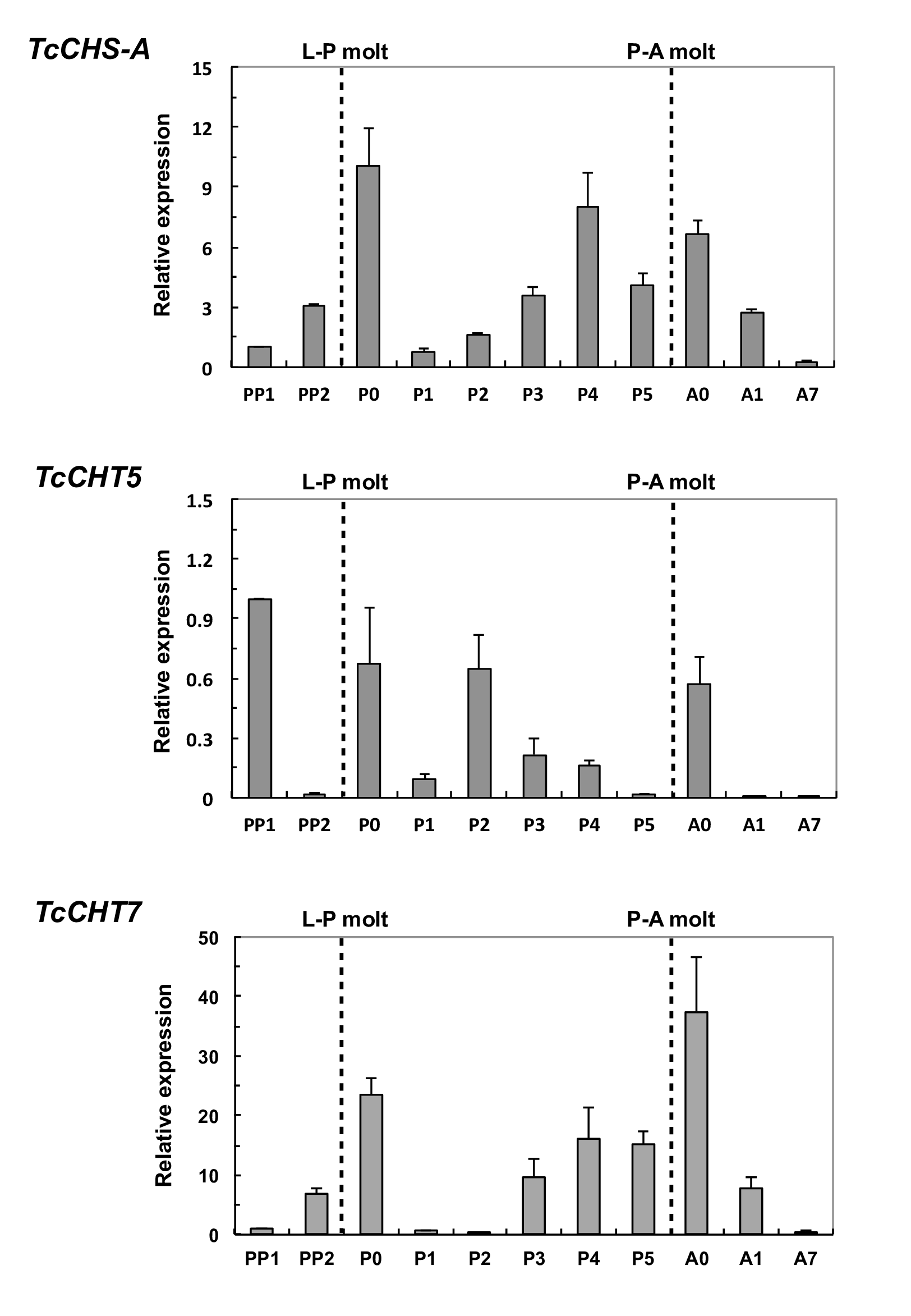

Supplement: S4 Fig — For the expression profiles of TcCHS-A and TcCHT5 by real-time PCR during late developmental stages, total RNA was extracted from whole insects between the early pharate pupal and young adult stages. PP1, 0–1 d-old pharate pupae; PP2, 1–2 d-old pharate pupae; P0, 0 d-old pupae; P1, 1 d-old pupae; P2, 2 d-old pupae; P3, 3 d-old pupae; P4, 4 d-old pupae; P5, 5 d-old pupae; A0, 0 d-old adults; A1, 1 d-old adults; A7, 7 d-old adults. Expression levels for TcCHS-A and TcCHT5 are presented relative to the levels of expression at the earliest developmental stage analyzed (PP1). See the legend of S3 Fig for details of expression analysis of TcCHT7 gene. All data are shown as the mean value ± SE (n = 3). (TIF) [file pgen.1007307.s007.tif]

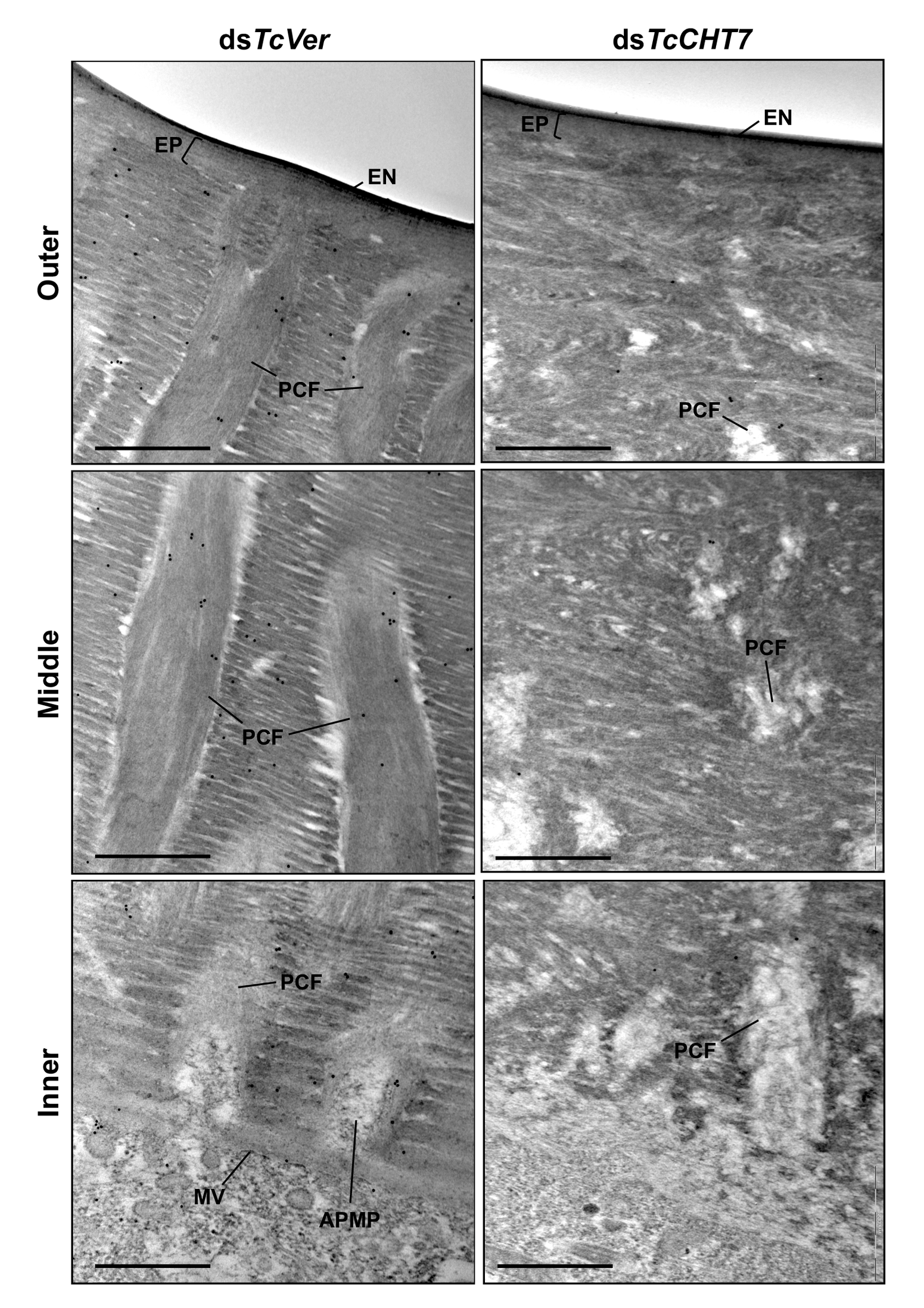

Supplement: S5 Fig — Ultra-thin sections of pharate adults (5 d-old pupae) that had been injected with dsRNA (200 ng per insect) for TcVer (left panels) and TcCHT7 (right panels) into the late instar larvae were incubated with anti-TcCHT7 antibody and washed extensively to remove unbound antibody. The antibody was then detected by goat anti-rabbit IgG conjugated to 10 nm gold particles. TcCHT7 protein is present in both horizontally oriented laminae and in vertical pore canal with pore canal fibers (PCF) in their core of the entire procuticle (exocuticle), but not in the envelope and epicuticle layers. The number of gold particles is obviously decreased in TcCHT7-deficient insects. EN, envelope; EP, epicuticle; MV, microvilli; APMP, apical plasma membrane protrusion. Scale bar = 500 nm. (TIF) [file pgen.1007307.s008.tif]

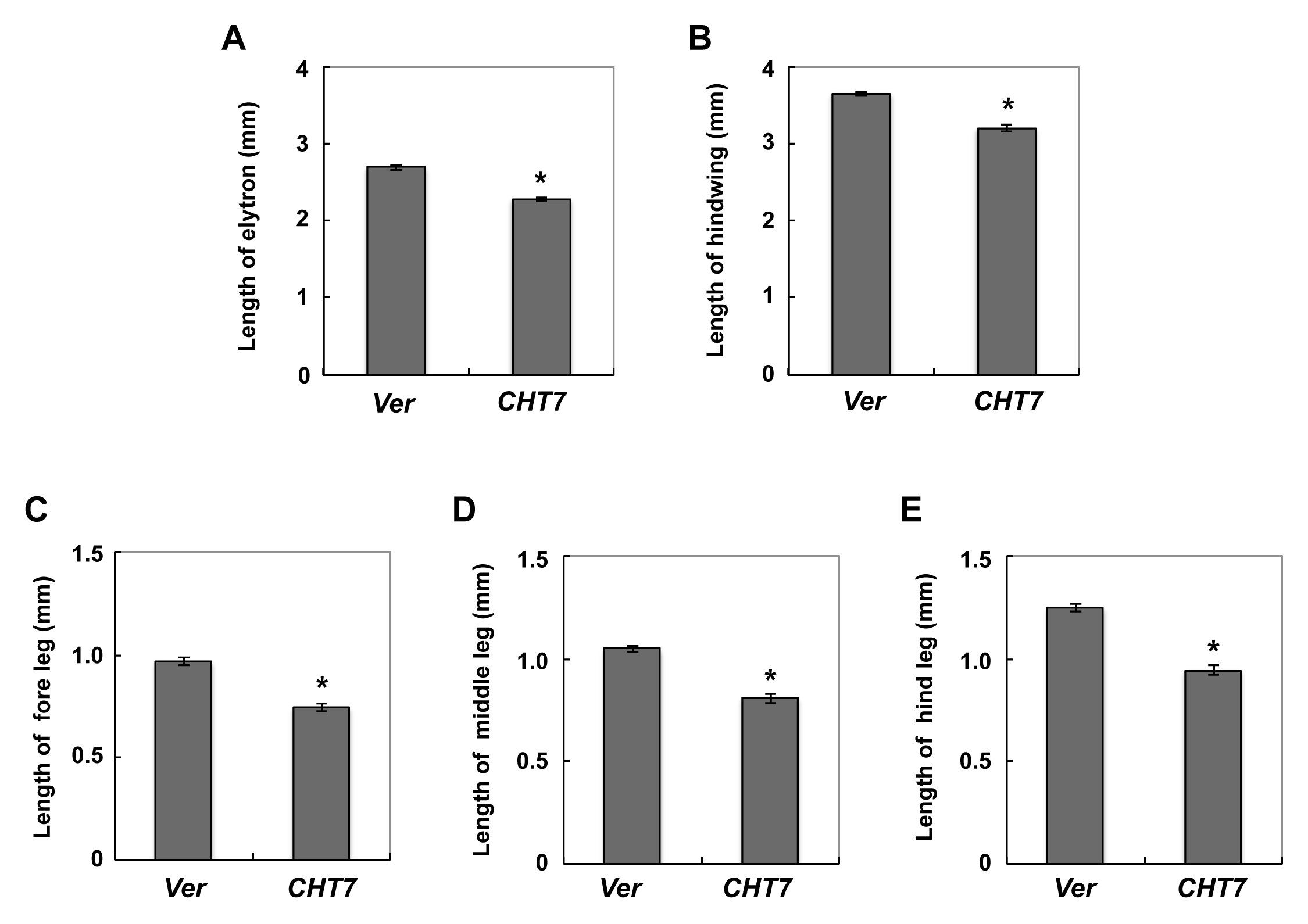

Supplement: S6 Fig — Elytra, hindwings and legs were dissected from 3–4 days old adults that had been injected with dsRNA for TcCHT7 (CHT7) and TcVer (Ver) in the late instar larvae. The length of the elytron (A), hindwing (B), fore leg (C), middle leg (D) and hind leg (E) were measured using ImageJ software. An asterisk indicates a significant difference in length between control and test insects (p < 0.0001, t-test). Data are shown as mean ± SE (n = 6–7). (TIF) [file pgen.1007307.s009.tif]

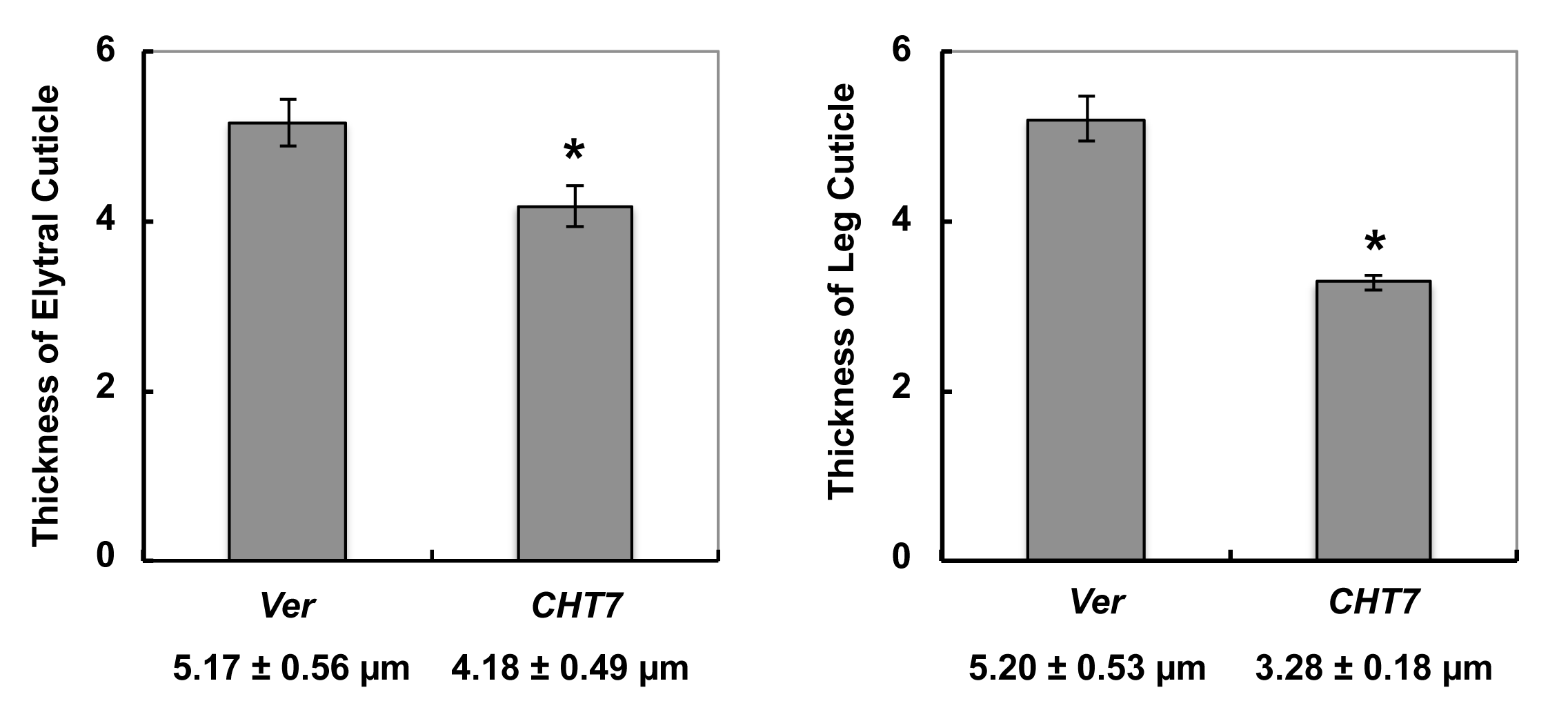

Supplement: S7 Fig — Ultrastructure of the elytral dorsal and leg cuticles from pharate adults (5 d-old pupae) that had been injected with dsRNA (200 ng per insect) for TcCHT7 (CHT7) and TcVer (Ver) at the late instar larval stage was analyzed by TEM followed by measurement of thickness of the cuticles. An asterisk indicates a significant difference in thickness between control and test insects (p < 0.03, t-test). Data are shown as mean ± SE (n = 6). (TIF) [file pgen.1007307.s010.tif]

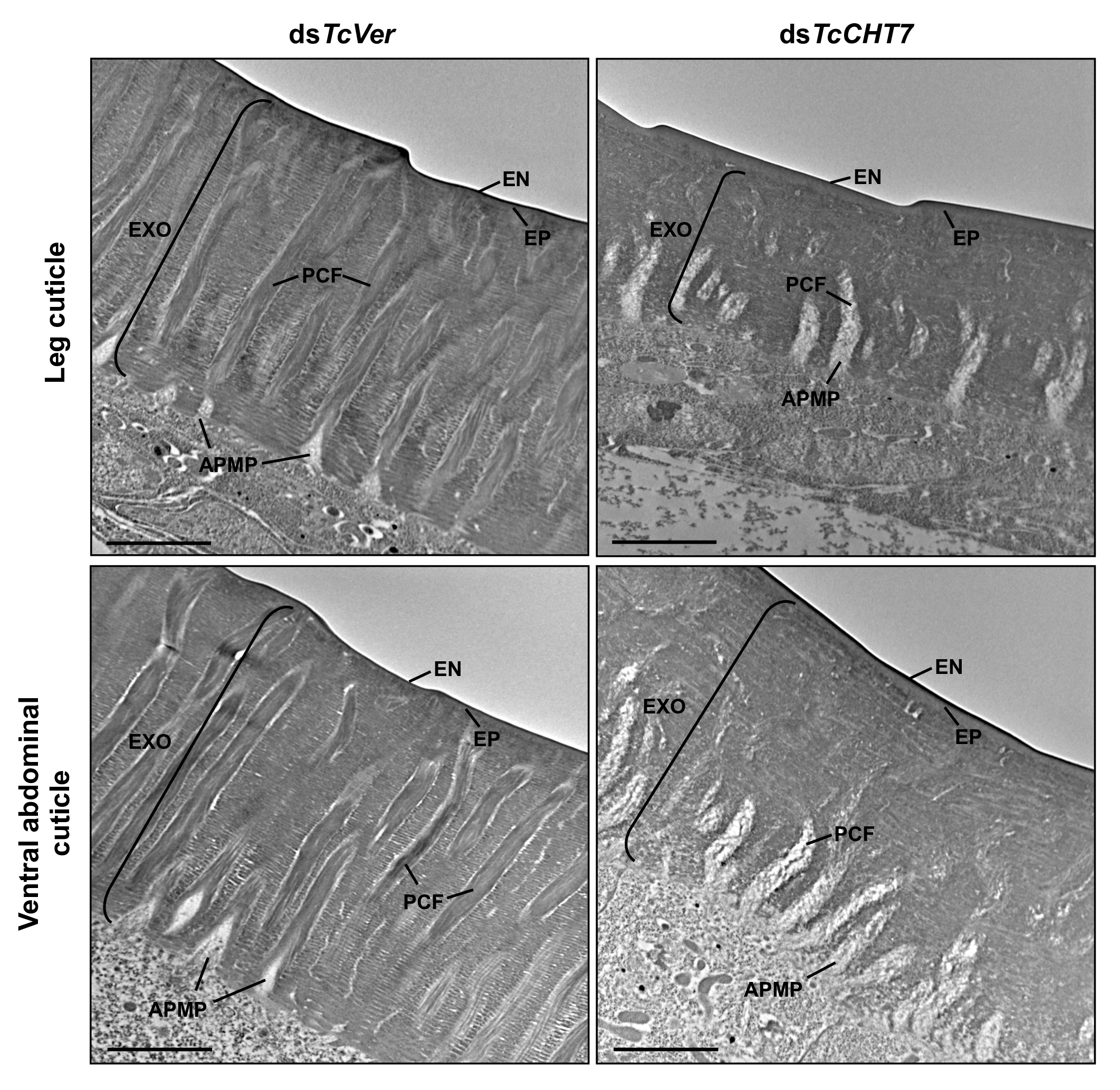

Supplement: S8 Fig — Ultrastructure of the rigid leg and ventral abdominal cuticles from pharate adults (5 d-old pupae) that had been injected with dsRNA (200 ng per insect) for TcVer and TcCHT7 at the late instar larval stage was analyzed by TEM. EN, envelope; EP, epicuticle; EXO, exocuticle; PCF, pore canal fiber; APMP, apical plasma membrane protrusion. Scale bar = 2 μm. (TIF) [file pgen.1007307.s011.tif]

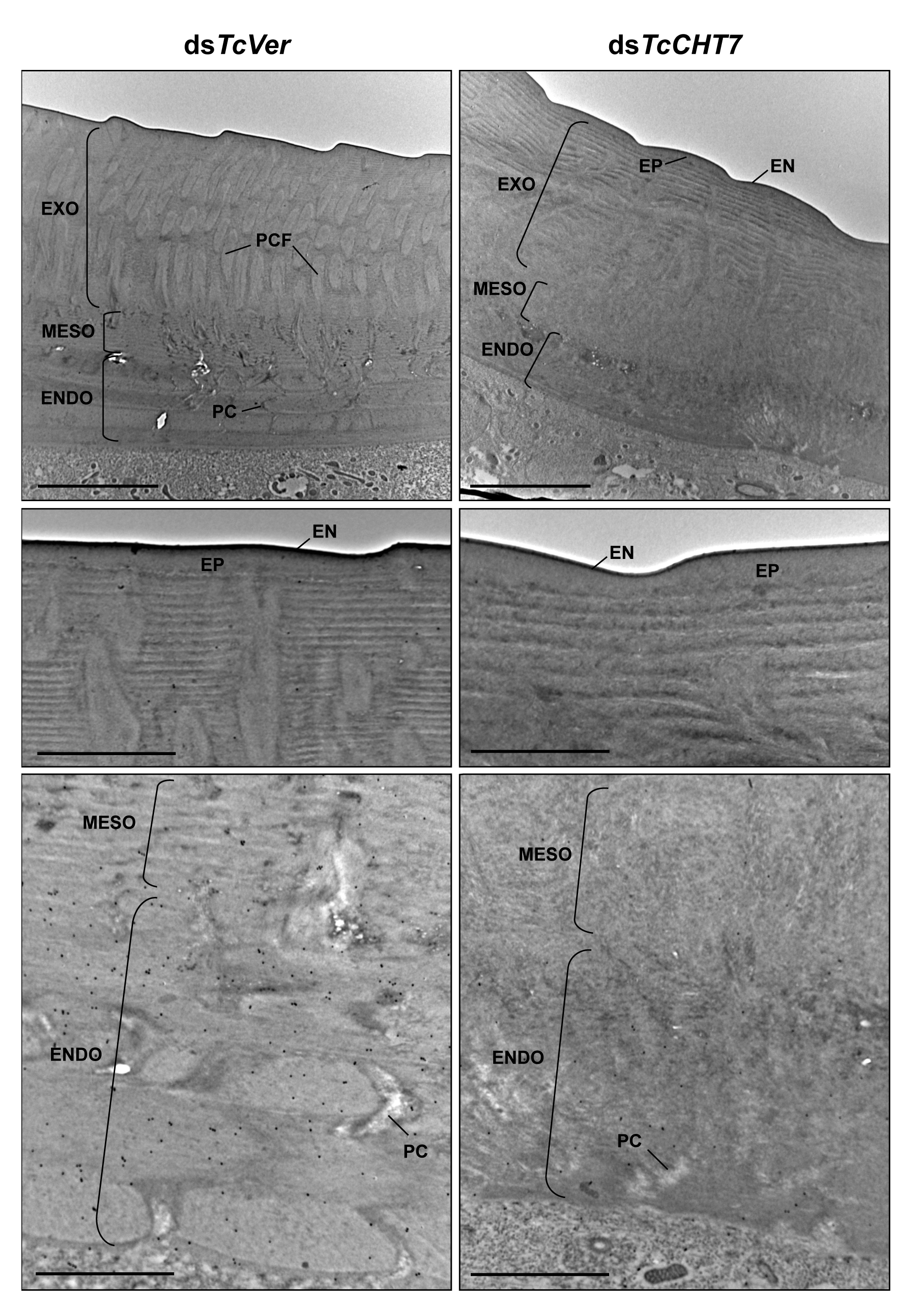

Supplement: S9 Fig — Ultra-thin sections of 3 d-old adults that had been previously injected with dsRNA (200 ng per insect) for TcVer (left panels) or TcCHT7 (right panels) at the late instar larvae were incubated with anti-TcCHT7 antibody, which was then detected by goat anti-rabbit IgG conjugated to 10 nm gold particles. TcCHT7 protein is present in the exocuticle (middle panels) as well as mesocuticle (MESO) and the endocuticle (ENDO) (bottom panels). The number of gold particles is obviously decreased in TcCHT7-deficient insects. EN, envelope; EP, epicuticle; PCF, pore canal fiber; PC, pore canal. (TIF) [file pgen.1007307.s012.tif]
